# Immunometabolic crosstalk in knee osteoarthritis: a bibliometric and translational analysis (2000–2025)

**DOI:** 10.3389/fimmu.2026.1877104

**Published:** 2026-09-17

**Authors:** Khalid Waleed, Ai Peng, Qirui Chen, Shenghao Cai, Yong Xia, Zuorui Zheng, Khalid Waqar, Ammara Arooj, Jiang Shan, Zhou Junwei, Jianjian Deng, Bin Zhou, Xiaoling Fu

**Affiliations:** 1 Department of Orthopedics, The Second Affiliated Hospital of Nanchang University, Nanchang, Jiangxi, China; 2 Jiangxi Medical College, Nanchang University, Nanchang, Jiangxi, China; 3 Collaboration Unit for the State Key Laboratory of Infectious Disease Prevention and Control, Nanchang, Jiangxi, China; 4 Center for Evidence-Based Medicine, Nanchang, Jiangxi, China; 5 Jiangxi Provincial Key Laboratory of Disease Prevention and Public Health, Nanchang, Jiangxi, China; 6 School of Public Health, Jiangxi Medical College, Nanchang University, Nanchang, Jiangxi, China; 7 Department of Cardiology, Punjab Institute of Cardiology, Lahore, Pakistan; 8 University College of Pharmacy, University of the Punjab, Lahore, Pakistan

**Keywords:** bibliometric analysis, immunometabolism, inflammatory signaling, innate immunity, knee osteoarthritis, metabolic dysfunction, translational medicine

## Abstract

**Background:**

Knee osteoarthritis (KOA) has traditionally been regarded as a localized degenerative joint disorder; however, accumulating evidence increasingly supports a critical role for immunometabolic dysfunction, chronic low-grade inflammation, and systemic metabolic alterations in disease initiation and progression. Although research in this multidisciplinary field is growing rapidly, the global intellectual structure, emerging mechanistic themes, and the evolution of the translation of KOA research remain inadequately documented.

**Methods:**

This study used a multi-database bibliometric and scientometric analysis with a hypothesis-generating translational synthesis, rather than a systematic review of intervention effects. We analyzed publications indexed in Web of Science, Scopus, and PubMed from 2000 to 2025. We used CiteSpace, VOSviewer, and Bibliometrix to analyze global research trends, collaboration networks, intellectual structure, and emerging themes in immunometabolic and inflammatory mechanisms in KOA.

**Results:**

A total of 783 publications were included. Annual research output increased substantially after 2015, reflecting growing recognition of systemic contributors to KOA. The United States and China were the largest contributors to the growth of international partnerships. Co-citation, keyword, and thematic analyses showed increasing research attention to metabolic syndrome, oxidative stress, innate immune activation, multimorbidity, and inflammatory signaling alongside traditional cartilage-focused research. Biomarker discovery, lifestyle intervention, phenotype stratification, and precision-intervention-focused therapies were emerging research hotspots, indicating the growing translational importance of metabolic and immune-mediated mechanisms in the pathogenesis of KOA.

**Conclusion:**

This analysis indicates increasing research attention to systemic immunometabolic factors alongside established joint-centered models of KOA. The identified research trajectories offer crucial translational insights and help inform the design of integrated approaches for risk stratification, prevention, and personalized management strategies involving targeting metabolic dysfunction, inflammatory signaling, and immune-mediated pathways in osteoarthritis.

## 1 Introduction

Knee osteoarthritis (KOA) is a common chronic musculoskeletal disorder, one of the leading causes of pain, disability, and functional impairments in the world. Rising life expectancy, aging of the population, and rising obesity and metabolic diseases have also played a major role in increasing the burden of osteoarthritis in the past few decades in most countries around the globe (1). Recent epidemiological estimates indicate that more than 500 million individuals worldwide are affected by osteoarthritis, with the knee joint being the most commonly affected (2, 3). Being one of the top causes of disability-adjusted life years (DALYs) in older adults, KOA has significant socioeconomic consequences on healthcare systems and societies, such as decreased work productivity, costly long-term treatment, and poor quality of life (4–6).

Osteoarthritis has traditionally been regarded as a degenerative joint disease primarily caused by mechanical wear and cartilage degeneration. Nevertheless, this has changed drastically over the last 20 years as evidence mounts to challenge this opinion. Accumulating data now support the view that KOA is a multifactorial pathology involving an intricate interplay among mechanical strain, age-related biological changes, metabolic imbalances, and inflammatory processes (5). Low-grade systemic inflammation has emerged as a key factor in disease onset and progression. Cytokines, adipokines, and acute-phase proteins are inflammatory mediators that may contribute to cartilage degradation, synovial inflammation, and subchondral bone remodeling (6). Moreover, other metabolic diseases such as obesity, diabetes, and metabolic syndrome are also believed to contribute to osteoarthritis, not only because of the mechanical loading of the joints but also because of the systemic inflammatory processes (7).

The increasing awareness of inflammation-related processes has prompted a growing body of evidence examining the interaction between systemic inflammation and KOA. Inflammatory biomarkers, metabolic phenotypes in osteoarthritis, and the role of multimorbidity in joint degeneration have been studied by many (8). In the meantime, new therapies targeting inflammatory pathways, including lifestyle, metabolic, and biological approaches, have begun to receive increasing attention in research (9). As a result, scientific knowledge on KOA and systemic inflammation has grown rapidly across disciplines such as orthopedics, rheumatology, metabolic medicine, and molecular biology.

Despite this growing body of research, researchers and clinicians still struggle to develop a holistic understanding of the intellectual framework, key players, and evolving subject matter of this interdisciplinary area. Traditional narrative reviews provide valuable insights yet tend to be less objective in their interpretation and fail to reflect the scope of the rapidly growing scientific literature (10). Bibliometric analysis is a quantitative technique for evaluating publication output, citation patterns, collaboration networks, and research trends, and for discovering new research topics in large datasets (11). Citation analysis and visualization can be combined with bibliometric techniques to get a clear view of how a research area has developed and of its knowledge structure (12).

In this study, we conducted a multi-database bibliometric and scientometric analysis of global research on KOA, systemic inflammation, and related metabolic factors. We used translational synthesis to contextualize the bibliometric findings and generate hypotheses, rather than to systematically evaluate treatment efficacy or establish causal mechanisms. Unlike previous broad osteoarthritis bibliometric studies, this analysis specifically maps the intersection of KOA with inflammatory, metabolic, and immunometabolic research across three databases and links the identified themes to a hypothesis-generating translational framework. The outcomes of this analysis may provide insight into the intellectual underpinnings of existing work and guide new research on inflammatory processes and their control in KOA (13).

## 2 Methods

### 2.1 Data sources and search strategy

We conducted a bibliometric and scientometric analysis of KOA-focused publications examining inflammatory, metabolic, or immunometabolic factors. Bibliometric techniques enable the quantitative assessment of the scale of scientific literature. They are often used to identify research trends, influential publications, and patterns of collaboration in fast-evolving research areas (14–16). We searched and exported records from Web of Science Core Collection (Clarivate; SCI-EXPANDED), Scopus (Elsevier), and PubMed (NCBI) on 15 February 2026. Searches covered publications from 1 January 2000 to 31 December 2025 and were restricted to English-language articles and reviews. The search combined KOA terms with inflammatory and metabolic concepts, including metabolic osteoarthritis or phenotype, systemic or low-grade inflammation, metabolic disorders, chronic systemic disease, multimorbidity, and cardiometabolic factors. Broad terms improved retrieval sensitivity, while we determined final eligibility based on substantive KOA relevance rather than term occurrence alone. We used database-specific search fields to improve search precision, such as TITLE-ABS-KEY fields in Scopus, topic searches in Web of Science, and MeSH terms and Title/Abstract searches in PubMed (17).

The database-specific search structures were: Scopus, TITLE-ABS-KEY((KOA terms) AND (metabolic/systemic inflammatory terms)); Web of Science, TS=((KOA terms) AND (metabolic/systemic inflammatory terms)); and PubMed, Knee Osteoarthritis MeSH or Title/Abstract terms combined with Metabolic Syndrome, Inflammation, or related Title/Abstract terms. **Supplementary File S1** provides the fully expanded equations, field tags, limits, and retrieved record counts. Although the retrieved dataset was restricted to knee osteoarthritis, broader osteoarthritis literature appeared in cited-reference networks when it contributed to the intellectual or mechanistic context of KOA research.

### 2.2 Eligibility criteria and data processing

We included English-language articles and reviews when KOA was the primary focus and an inflammatory, metabolic, or immunometabolic factor was substantively examined. Studies containing only incidental mentions of KOA or these factors were excluded, along with conference proceedings, book chapters, early-access records, and records with incomplete bibliographic data. All three databases contributed core publication metadata. Web of Science and Scopus supplied citation counts and cited-reference data for citation-dependent analyses, whereas PubMed additionally contributed PMID and MeSH data. We used PubMed-only records lacking citation or cited-reference data for descriptive, collaboration, and keyword analyses; the software automatically omitted records missing the metadata required for each analysis.

After merging the datasets, we removed duplicate records using CiteSpace’s built-in deduplication function, which compares core bibliographic fields (author, title, journal, DOI, and publication year) and retains the more complete record. We also checked residual duplicate, invalid, and ineligible records during title and abstract screening.

This was a bibliometric and scientometric study, not a systematic review or meta-analysis of clinical outcomes. We used the PRISMA 2020 flow-diagram structure only to report the literature identification, deduplication, screening, and inclusion process transparently. We provide a completed PRISMA 2020 checklist, with non-applicable systematic-review items identified accordingly, as **Supplementary File S3**. After database searches, dataset merging, and removal of duplicate and ineligible records, the final harmonized dataset comprised 783 unique eligible publications and was used for all analyses, including the country analysis; no 751-record subset was used (**Figure 1**).

**Figure 1:**
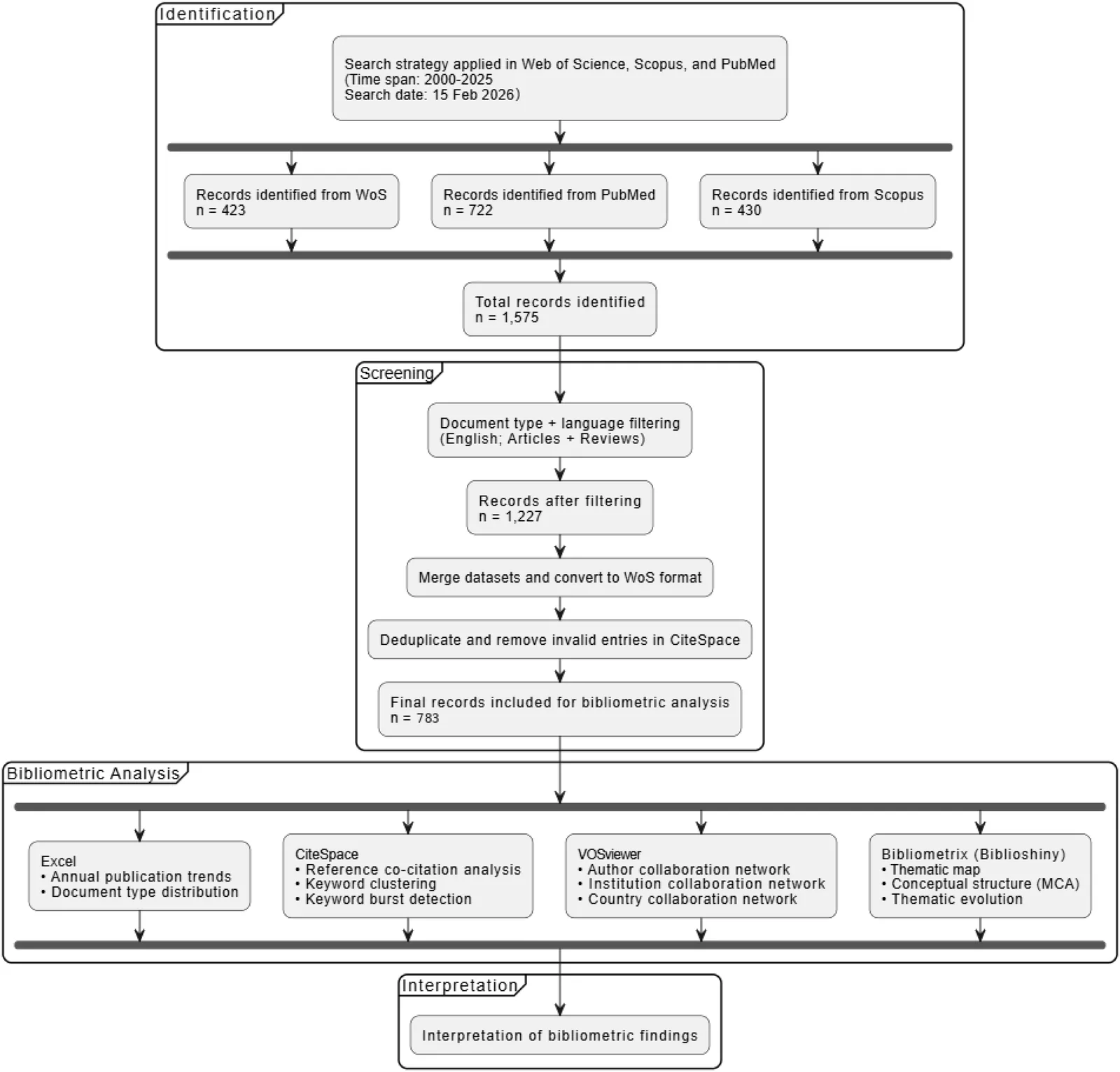
Workflow of literature retrieval, screening, and bibliometric analysis for studies on knee osteoarthritis and systemic inflammation/metabolic factors (2000–2025). The diagram illustrates the process of database searching, record screening, duplicate removal, and final inclusion of eligible studies according to predefined criteria.

### 2.3 Bibliometric analysis

To comprehensively explore the intellectual structure and research trends within the field, three complementary bibliometric tools were used: CiteSpace (version 6.4.R1), VOSviewer (version 1.6.20), and the Bibliometrix R package (version 4.1.4; Biblioshiny interface). CiteSpace was primarily used to analyze the knowledge base and detect new research hotspots through reference co-citation analysis, keyword co-occurrence clustering, and burst identification, thereby identifying influential studies and emerging research themes in a rapidly developing state (18). Country collaboration was visualized using VOSviewer, whereas institutional and author collaboration networks were analyzed using CiteSpace (19). They conducted thematic and conceptual literature analysis using Bibliometrix, including thematic mapping and analysis of the conceptual structure of research topics (20). Before the revised burst and thematic analyses, we harmonized keywords using a rule-based procedure. We merged synonymous variants, excluded database-indexing artifacts and nonspecific measurement terms, and retained clinically meaningful terms. **Supplementary Table S1** provides the complete synonym and exclusion lists.

### 2.4 Visualization parameters

CiteSpace time slicing was 2000-2025, with a 1-year slice, and the node selection criterion was the g-index (k = 25). We cleaned and harmonized keywords before analysis according to the rules reported in **Supplementary Table S1**. Keyword bursts were detected using the harmonized Author Keywords and Keywords Plus fields, and the 20 strongest bursts were visualized. We generated the thematic map from 198 harmonized Author Keywords using a minimum cluster frequency of 5 per 1,000 documents and an alpha value of 0.5. For the VOSviewer country-collaboration network, we used full counting, a minimum threshold of five publications per country, association-strength normalization, and the default clustering and layout settings. Complete analysis-specific thresholds and network-construction settings are provided in **Supplementary File S2**.

We used cosine strength as a network-visualization parameter. We used the Pathfinder and pruning-sliced network algorithms to prune the networks, improve clarity, and highlight the most significant relationships among nodes. We considered various node types, including references, keywords, authors, institutions, and countries, enabling identification of the intellectual background, collaborations, and emerging research themes in knee osteoarthritis and systemic inflammation. We detail the parameter settings and provide reproducibility information in **Supplementary File S2**.

### 2.5 Sensitivity analysis

Sensitivity analyses repeated the keyword-network analysis after excluding 2025 publications, restricting the dataset to articles, and restricting the dataset to Web of Science records. We applied the same network construction, keyword harmonization, and pruning settings as in the primary analysis (**Supplementary Table S2**).

## 3 Results

### 3.1 Rapid expansion of research on KOA and systemic inflammation

We included 783 publications on knee osteoarthritis and systemic inflammatory or metabolic factors after merging records from Web of Science Core Collection, Scopus, and PubMed and removing duplicates and ineligible records. Publication pattern in terms of time is depicted in **Figure 2A**, and the average pattern of citations per year is represented in **Figure 2B**.

**Figure 2:**
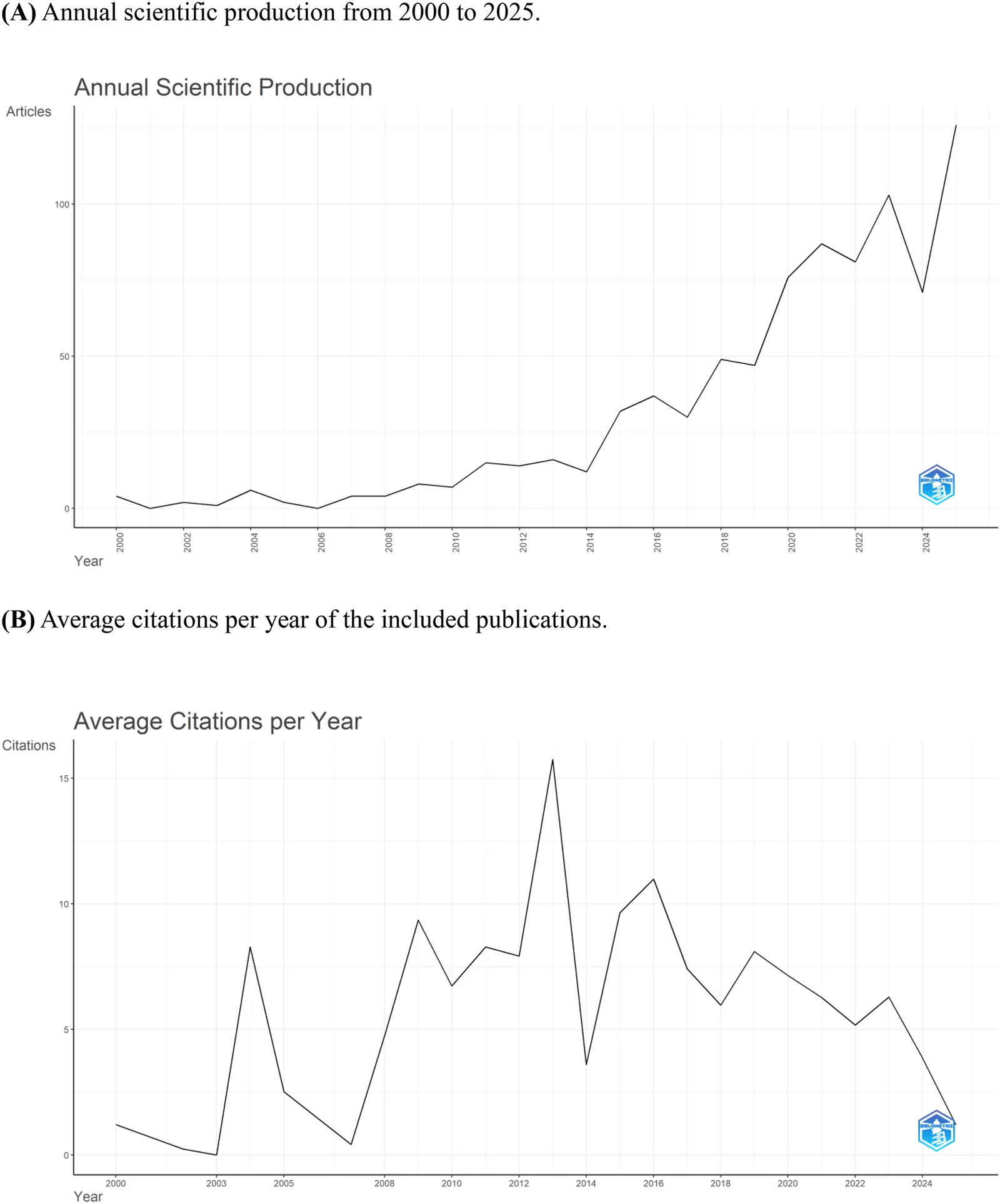
Temporal trends in scientific production and citation impact in research on knee osteoarthritis and systemic inflammation. **(A)** Annual scientific production from 2000 to 2025. **(B)** Average citations per year of the included publications. Visualizations were generated using the Bibliometrix package in R.

Based on observed changes in annual publication output and thematic evolution, the field’s development can be broadly divided into three phases: early (2000–2010), middle (2011–2018), and recent (2019–2025). In the early years (2000-2010), research was relatively inactive, with annual publications not exceeding 10, and limited research attention was directed toward systemic inflammation and metabolic causes of knee osteoarthritis. Since around 2011, the volume of publications has grown steadily, with a significant increase after 2015. Annual publication output reached 129 publications in 2025, the highest annual total during 2000–2025.

The number of citations per year was quite volatile; the highest levels were observed in the early to mid-2010s. The peaks likely reflect the release of major works that paved the way for linking inflammation and metabolic dysfunction to osteoarthritis. On the other hand, the relatively small average number of citations in more recent years may reflect a shorter time horizon for newly published research to gain citations, rather than a decrease in research impact.

### 3.2 Global research is concentrated in high-income countries, with emerging contributions from Asia

A total of 783 publications on systemic inflammation, metabolic factors, and knee osteoarthritis (KOA) were contributed by authors from multiple countries and regions worldwide. The United States produced the most publications (198, 25.29%), followed by China (128, 16.35%) and Iran (64, 8.17%). Other significant sources were Brazil, Italy, England, the Netherlands, Canada, India, and South Africa (**Table 1**).

**Table 1:** Most productive countries and their collaboration characteristics in research on knee osteoarthritis and systemic inflammation.

Rank	Country	Articles	Articles (%)	TC	AC	SCP	MCP	MCP (%)	Centrality
1	United States	198	25.29	12432	62.8	145	53	26.77	0.37
2	China	128	16.35	5631	44.0	102	26	20.31	0.18
3	Iran	64	8.17	1840	28.7	48	16	25.00	0.09
4	Brazil	48	6.13	1734	36.1	33	15	31.25	0.12
5	Italy	42	5.36	2215	52.7	28	14	33.33	0.15
6	England	39	4.98	2568	65.8	22	17	43.59	0.21
7	Netherlands	39	4.98	2387	61.2	21	18	46.15	0.19
8	Canada	39	4.98	1974	50.6	24	15	38.46	0.16
9	India	36	4.60	842	23.4	27	9	25.00	0.07
10	South Africa	34	4.34	913	26.9	20	14	41.18	0.11

Data source: article counts, percentages, and collaboration indicators were calculated from the merged and deduplicated Web of Science, Scopus, and PubMed dataset (n = 783). Total and average citation counts were derived from citation data available in Web of Science and Scopus.

TC, total citations; AC, average citations per article; SCP, single-country publications; MCP, multiple-country publications; Centrality, betweenness centrality from collaboration network.

The United States also showed the highest citation impact, with 12,432 citations, and the highest total research influence (average citations [AC] = 62.8). England and the Netherlands had high average citation rates (AC = 65.8 and 61.2, respectively), and Italy and Canada also showed strong citation performance, suggesting a significant impact of international research.

Patterns of international cooperation differed significantly among countries. The United States had the largest number of multi-country publications (MCP = 53), reflecting extensive international collaboration. Integration was high in European countries, particularly in the Netherlands and England, with the highest international collaboration rates (MCPI = 46.15% and 43.59%, respectively). There was also active participation of Canada and South Africa in multinational research networks.

The collaboration network analysis also revealed the structure of the global scientific collaboration network (**Figure 3A**). The United States, China, England, and the Netherlands showed comparatively high betweenness centrality, indicating stronger bridging positions within the international collaboration network. **Figure 3B** illustrates the distribution of research and collaboration activities worldwide. Newly emerging countries, such as Iran, India, and Brazil, demonstrated increasing publication output but lower centrality values.

**Figure 3:**
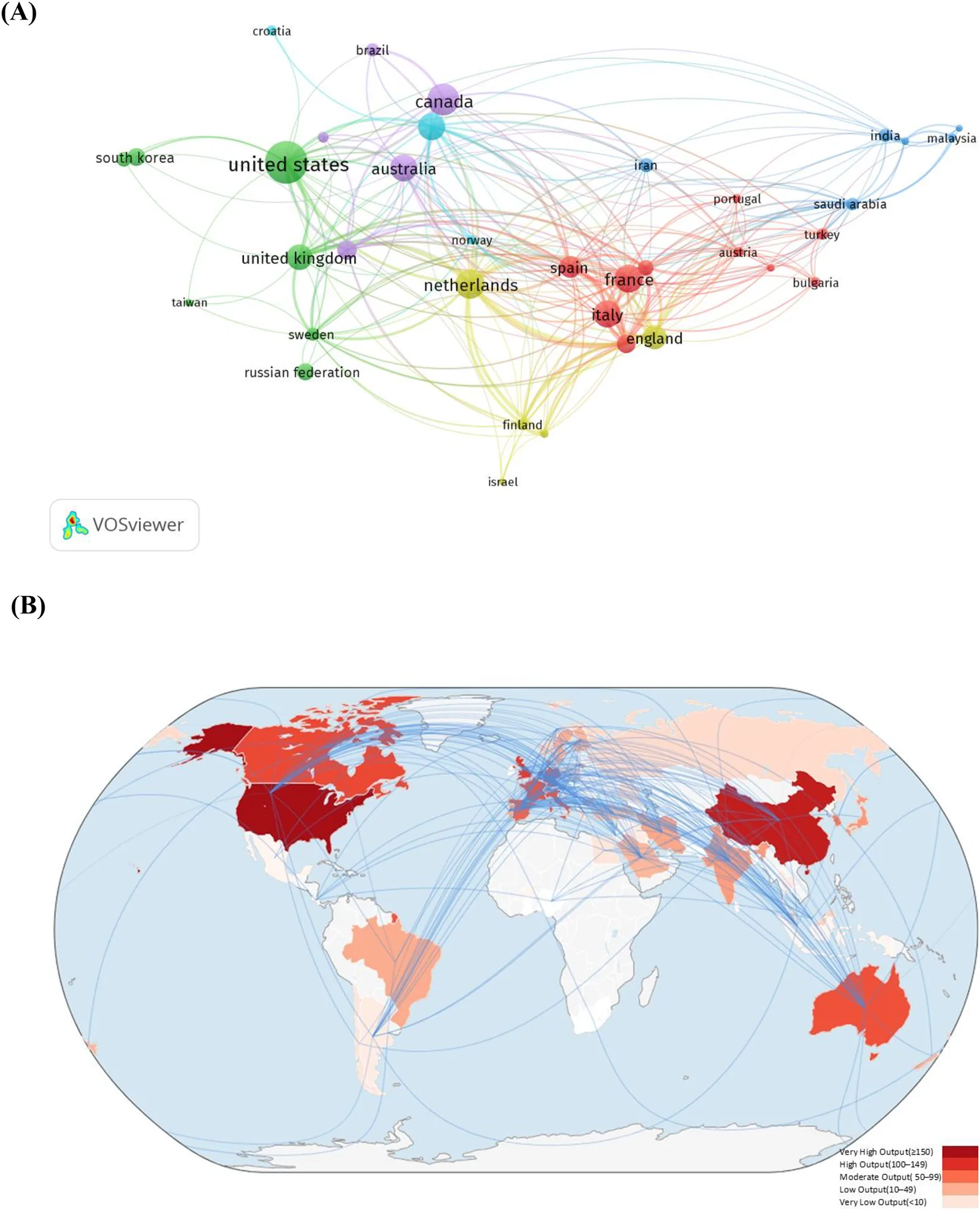
**(A)** International collaboration network among countries in research on knee osteoarthritis and systemic inflammation. An international collaboration network among countries. Node size represents publication output, and link thickness indicates collaboration strength. The network was generated in VOSviewer using full counting, a minimum threshold of five publications per country, and association-strength normalization. **(B)** Global geographic distribution of publications and international research collaboration. Country shading represents publication output, while connecting lines indicate international collaborative relationships.

Overall, global production in this area remains concentrated in high-income countries, especially the United States and some European countries. Still, in recent years, publication activity has grown significantly in emerging areas.

### 3.3 Research activity is concentrated among a limited number of institutions and author clusters

Institutional analysis revealed that research on systemic inflammation and metabolic dysfunction, and knee osteoarthritis (KOA) was concentrated within a comparatively small number of very productive academic institutions. Several institutions showed consistently high publication output, citation counts, and network connectivity. Institutional and author productivity results are presented in **Tables 2A, B**, respectively.

**Table 2A:** Leading institutions contributing to research on knee osteoarthritis and systemic inflammation.

Rank	Institution	Country	Articles	Centrality
1	Boston University	USA	12	0.13
2	Harvard Medical School	USA	11	0.12
3	University Medical Center Utrecht	Netherlands	10	0.11
4	University of Oxford	UK	9	0.10
5	University of California, San Francisco	USA	9	0.09
6	Duke University	USA	8	0.08
7	Wake Forest University	USA	8	0.07
8	Sun Yat-sen University	China	7	0.06
9	University of Melbourne	Australia	7	0.05
10	University of Tasmania	Australia	6	0.04

Data source: institutional publication counts and collaboration-network centrality were calculated from the merged and deduplicated Web of Science, Scopus, and PubMed dataset (n = 783).

**Table 2B:** Leading authors by publication and citation indicators in research on knee osteoarthritis and systemic inflammation.

Rank	Author	Country	Articles	TC	h-index	g-index	PY start
1	Kraus VB	USA	22	1540	16	28	2005
2	Berenbaum F	France	18	1482	15	26	2004
3	Sellam J	France	15	1106	14	23	2007
4	Mobasheri A	UK	14	980	13	21	2008
5	Griffin TM	USA	13	945	12	20	2009
6	Guilak F	USA	12	902	12	19	2006
7	Samuels J	USA	10	740	11	18	2010
8	Liu Y	China	10	602	9	15	2012
9	Zhang Z	China	9	575	9	14	2013
10	Wang J	China	9	560	8	13	2014

Data source: author publication counts were calculated from the merged and deduplicated Web of Science, Scopus, and PubMed dataset (n = 783). Total citations, h-index, and g-index were calculated using citation data available in Web of Science and Scopus.

Network analysis of collaboration showed distinct clusters of institutional collaboration (**Figure 4A**). The top-ranked institutions were mainly from the U.S., China, and some European countries, with higher collaborative connectivity. Institutions with higher betweenness centrality occupied stronger bridging positions between research groups and international collaborations.

**Figure 4:**
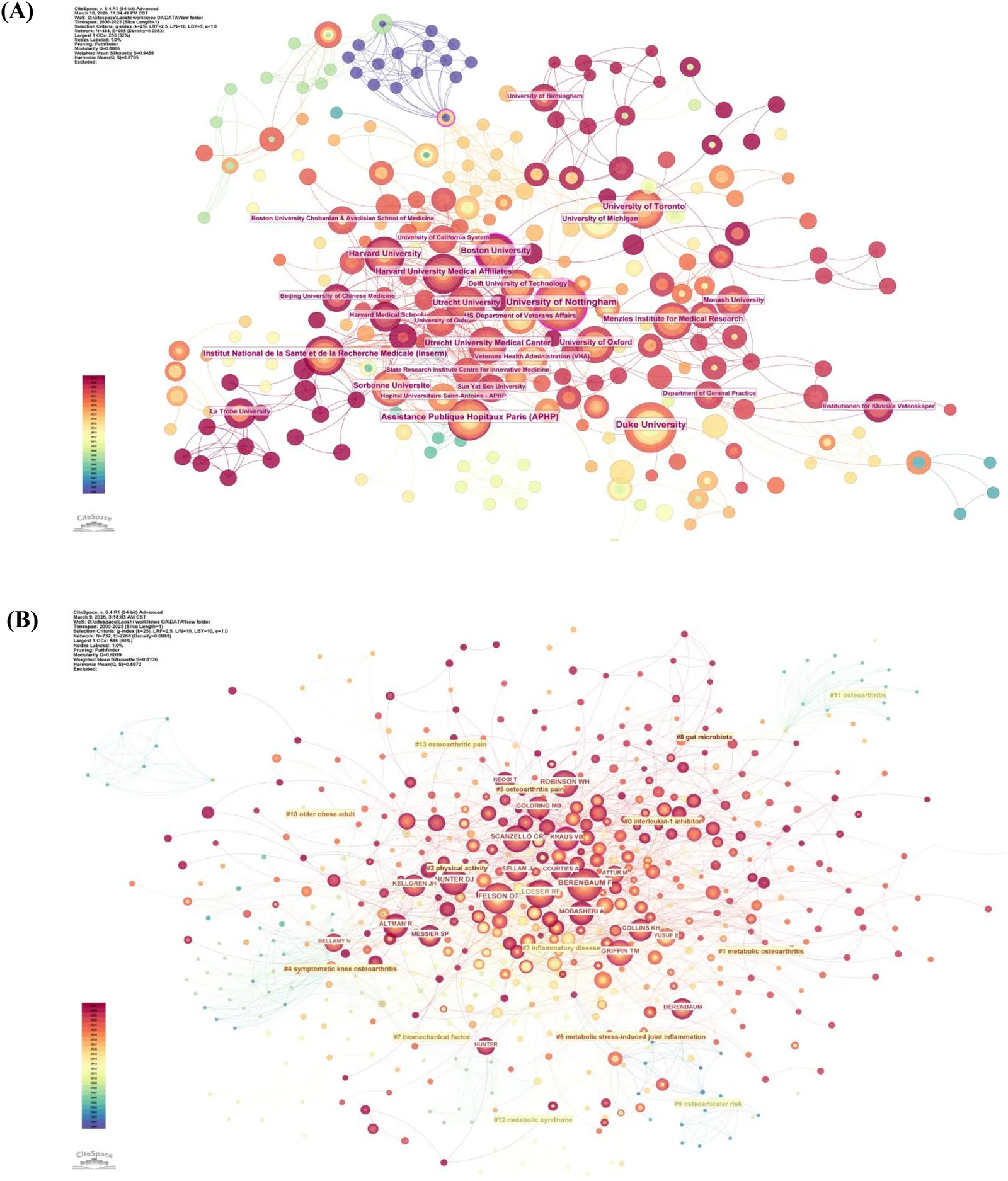
**(A)** Institutional collaboration network in knee osteoarthritis research. Nodes represent institutions, node size reflects publication output, and links indicate collaborative relationships. The network was generated in CiteSpace using one-year time slices, g-index selection (k = 25), cosine link strength, and Pathfinder pruning. The network contained 484 nodes and 965 links (density = 0.0083; modularity Q = 0.8065; mean silhouette S = 0.9455). **(B)** Author co-authorship network in knee osteoarthritis research. Nodes represent authors, node size reflects publication output, and links indicate co-authorship relationships. We generated the network in CiteSpace using one-year time slices, g-index selection (k = 25), cosine link strength, and Pathfinder pruning. The network contained 732 nodes and 2,268 links (density = 0.0085; modularity Q = 0.8136; mean silhouette S = 0.6972).

Author analysis also revealed that the research field was included several high-output and highly cited author groups that collaborated closely (**Figure 4B**). A limited number of authors contributed disproportionately to publication output and citation impact, forming closely connected research clusters focused on inflammation, metabolic dysfunction, biomarkers, and osteoarthritis progression.

Co-authorship mapping revealed several active collaboration networks, but co-authorship remained relatively concentrated within institutional and regional groups. However, several authors with high citation influence and network connectivity played a large role in shaping the field’s intellectual structure.

Overall, the results show that in this field, research activity is concentrated in a relatively small set of institutions and author groups with comparatively high publication output, citation counts, and network connectivity with high international visibility and research impact.

### 3.4 Research dissemination and knowledge integration are dominated by a small number of core journals

Journal distribution analysis demonstrated that publications on systemic inflammation, metabolic dysfunction, and knee osteoarthritis (KOA) were concentrated in a relatively small set of core journals, despite spanning multiple academic fields. This trend suggests a highly concentrated publication structure in the field of research.

Bradford’s Law analysis identified a small group of journals responsible for a substantial proportion of total scientific output (**Figure 5A**). The most productive journal in this category was Osteoarthritis and Cartilage (46 publications), followed by the International Journal of Molecular Sciences and Scientific Reports (**Table 3**). Other sources were specialized rheumatology journals, such as Arthritis Research and Therapy and Clinical Rheumatology.

**Figure 5:**
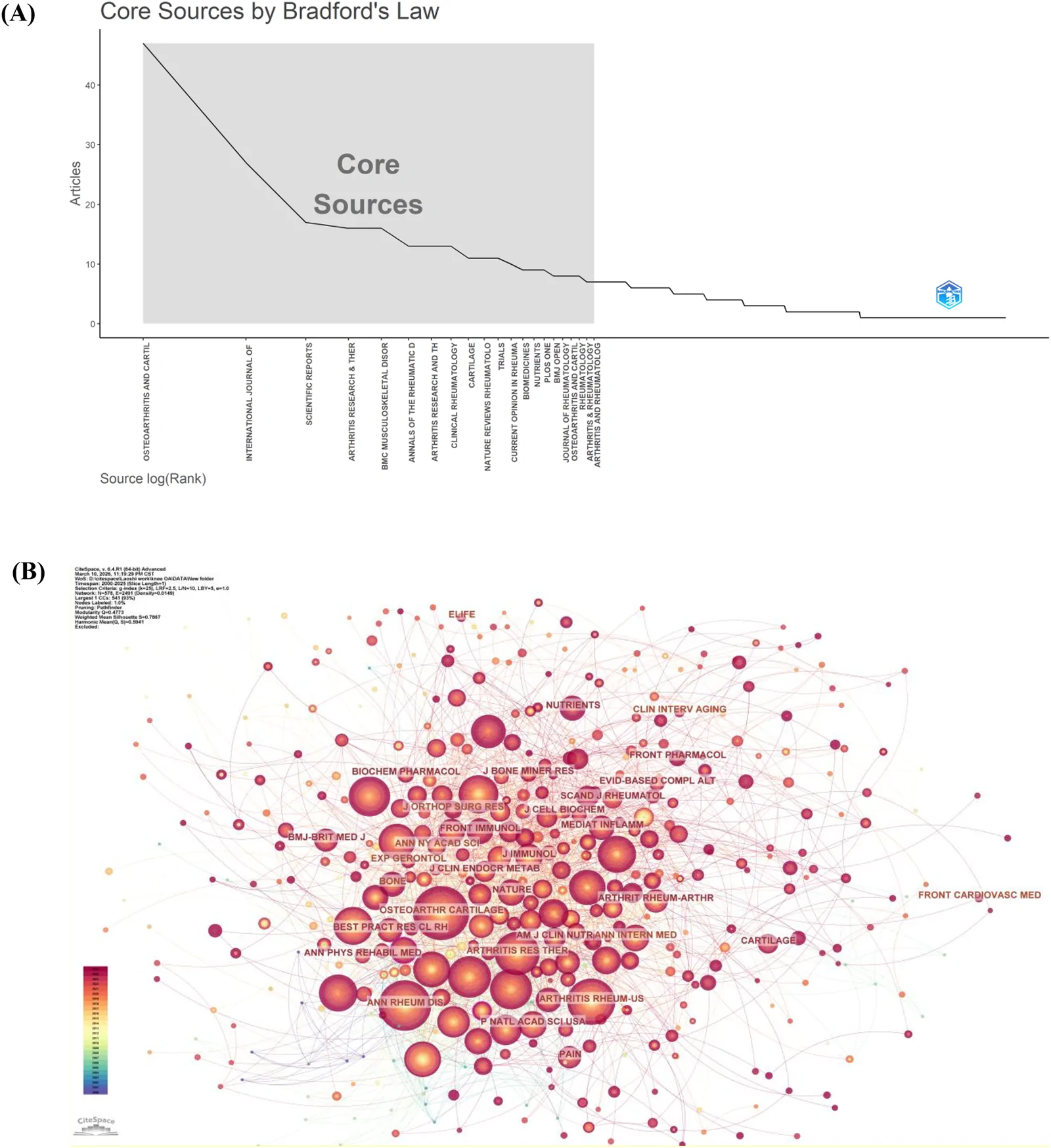
**(A)** Core sources identified using Bradford’s Law. The Bradford distribution illustrates the concentration of publications among a limited set of core journals that publish most articles in the research field. **(B)** Journal co-citation network in research on knee osteoarthritis and systemic inflammatory or metabolic factors. Nodes represent journals, node size reflects co-citation frequency, and links indicate co-citation relationships between journals. The visualization was generated using CiteSpace. The CiteSpace network used one-year time slices, g-index selection (k = 25), cosine link strength, and Pathfinder pruning. It contained 758 nodes and 2,491 links (density = 0.0149; modularity Q = 0.4730; mean silhouette S = 0.7867).

**Table 3:** Leading journals publishing research on knee osteoarthritis and systemic inflammatory or metabolic factors.

Rank	Journal	Country	Articles	TC	h-index	g-index	PY start
1	Osteoarthritis and Cartilage	UK	46	2875	22	34	2002
2	International Journal of Molecular Sciences	Switzerland	27	1015	15	22	2015
3	Scientific Reports	UK	17	430	9	13	2016
4	Arthritis Research & Therapy	UK	16	894	13	18	2004
5	BMC Musculoskeletal Disorders	UK	16	375	9	14	2007
6	Clinical Rheumatology	Germany	13	248	7	10	2006
7	Annals of the Rheumatic Diseases	UK	12	1560	14	22	2003
8	Arthritis & Rheumatology	USA	11	1240	12	18	2001
9	Journal of Clinical Investigation	USA	10	1310	11	17	2004
10	Nature Reviews Rheumatology	UK	9	980	10	15	2011

Data source: Journal publication counts were calculated from the merged and deduplicated Web of Science, Scopus, and PubMed dataset (n = 783). Total citations, h-index, and g-index were calculated using citation data available in Web of Science and Scopus.

TC, total citations; PY start, first publication year in this dataset.

Citation analysis showed that some high-impact rheumatology journals held dominant positions within the field’s body of knowledge. The highest citation influence was observed in Annals of the Rheumatic Diseases and Arthritis and Rheumatology, both highly cited sources within the analyzed literature.

The intellectual structuring of the literature was also reflected in a co-citation network analysis of journals (**Figure 5B**). The most frequently cited journals were at the center of the network and served as key hubs for knowledge integration across osteoarthritis, inflammation, metabolism, and translational research.

Overall, a comparatively small number of journals dominated knowledge distribution and absorption in this area, with high citation counts and strong network connectivity in the scientific cooperation network.

### 3.5 The intellectual structure of KOA research is organized around metabolic–inflammatory–cartilage interactions

CiteSpace and VOSviewer were used to conduct reference co-citation and keyword analyses to assess the intellectual structure of the body of research on knee osteoarthritis (KOA) and its relation to systemic inflammation and metabolic dysfunction. The reference co-citation network (**Figure 6A**) had 739 nodes, 2,044 links, and a network density of 0.0075. Cluster analysis showed high structural reliability, with a modularity value (Q) of 0.7808 and a mean silhouette score (S) of 0.8779, indicating a stable, well-defined knowledge structure. The significant thematic groups were mechanistic insights, metabolic influences, new pharmacological treatments, chondrocyte activation, and osteoarthritis related to metabolic syndrome.

**Figure 6:**
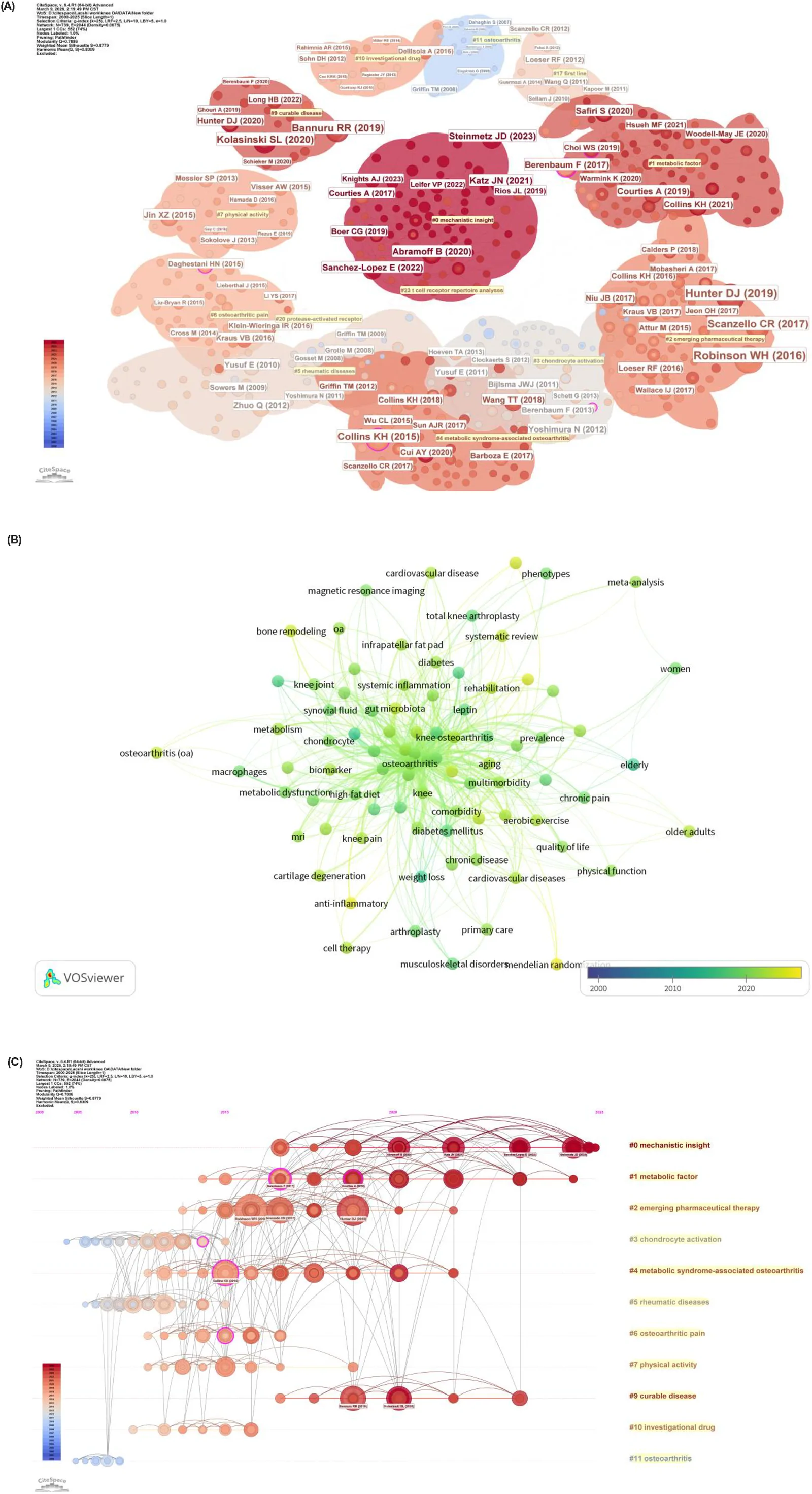
**(A)** Reference co-citation network illustrating the intellectual structure of KOA immunometabolic research. Nodes represent cited references, and node size reflects citation frequency. Links indicate co-citation relationships between references. The CiteSpace network used one-year time slices, g-index selection (k = 25), cosine link strength, and Pathfinder pruning. It contained 739 nodes and 2,044 links (density = 0.0075; modularity Q = 0.7808; mean silhouette S = 0.8779). **(B)** Keyword co-occurrence network illustrating major research themes. Nodes represent keywords, and node size reflects occurrence frequency. The overlay network was generated in VOSviewer using full counting and association-strength normalization. Node size represents keyword occurrence, link thickness indicates co-occurrence strength, and node color represents the average publication year. **(C)** Timeline visualization of keyword clusters showing the evolution of research themes over time. Each horizontal line represents a research cluster, and nodes represent keywords appearing during specific time periods. The timeline illustrates the emergence, development, and persistence of major research topics across the study period.

Frequently co-cited works by Loeser RF, Robinson WH, Scanzello CR, and Hunter DJ occupied highly connected positions in the co-citation network. These studies contributed substantially to the conceptual framework of integrating inflammatory signaling, metabolic dysfunction, immune-based processes, and cartilage degeneration in osteoarthritis studies.

The analysis of keyword co-occurrence also indicated how the field is conceptually organized (**Figure 6B**). Keywords that recurred included osteoarthritis, knee osteoarthritis, systemic inflammation, aging, multimorbidity, diabetes mellitus, biomarker, chondrocyte, synovial fluid, and gut microbiota, reflecting growing research interest in the interactions among metabolic dysfunction, inflammatory signaling, immune-mediated processes, and cartilage biology.

The temporal development of research themes was illustrated through the timeline visualization of keyword clusters (**Figure 6C**). Initial research put much emphasis on the pathology of osteoarthritis in general and the mechanisms of rheumatic diseases. Subsequent studies focused more on metabolic syndrome-related osteoarthritis, inflammatory mechanisms, oxidative stress, and chondrocyte biology. More recent themes included mechanistic knowledge, investigational therapies, physical exercise-based interventions, and translational disease models, and suggested a progressive transition toward integrative and immunometabolic research models.

Overall, the literature increasingly connected metabolic dysfunction, inflammatory signaling, immune-related processes, and cartilage degeneration as interrelated research themes in KOA.

### 3.6 Research hotspots have shifted toward a multidimensional metabolic, inflammatory, and translational framework

Keyword burst detection, thematic mapping, and dual-map overlay analyses were performed using CiteSpace and Bibliometrix to identify hotspots and evolving trends in knee osteoarthritis (KOA) research. Keyword burst analysis identified terms that increased sharply during specific periods, revealing shifts in research focus over time.

Early bursts included arthritis, C-reactive protein, leptin, adiponectin, and rheumatoid arthritis. During the intermediate period, risk factors, hand osteoarthritis, pathology, immunology, disease course, and adipose tissue became prominent (**Figure 7**).

**Figure 7:**
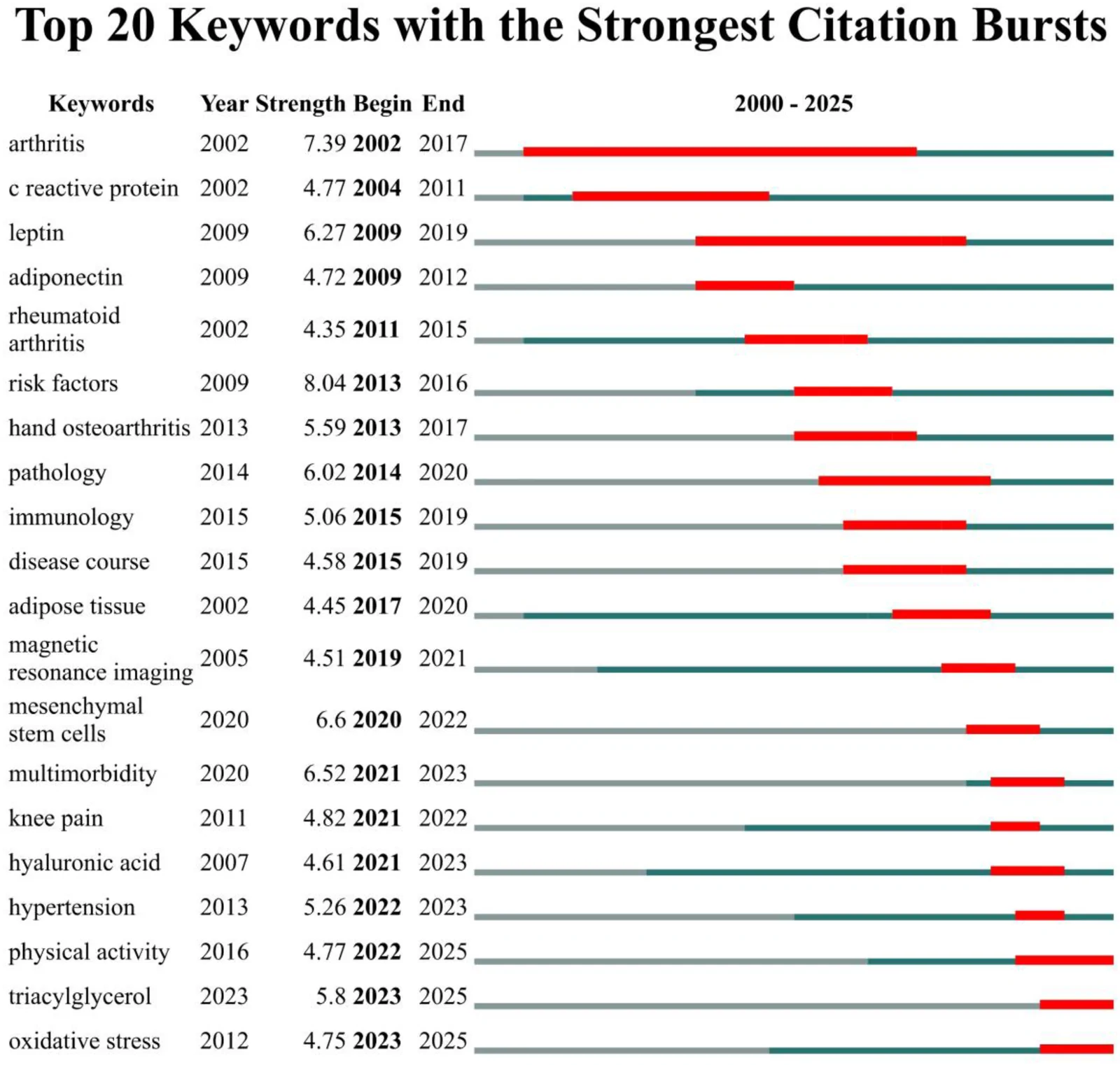
Top 20 keywords with the strongest citation bursts from 2000 to 2025. Red segments indicate periods of heightened keyword activity after keyword harmonization.

Recent bursts included multimorbidity, knee pain, hyaluronic acid, hypertension, physical activity, triacylglycerol, and oxidative stress. Physical activity, triacylglycerol, and oxidative stress remained active through 2025, indicating sustained attention to lifestyle and metabolic mechanisms.

The thematic map identified osteoarthritis, inflammation, and pain as central basic themes, together with obesity, metabolic syndrome, adipokines, multimorbidity, and comorbidity (**Figure 8**). Knee osteoarthritis, gut microbiota, and systemic inflammation represented a developing interconnected theme, while low-grade inflammation, treatment, rehabilitation, aging, macrophages, and fibrosis formed more specialized themes.

**Figure 8:**
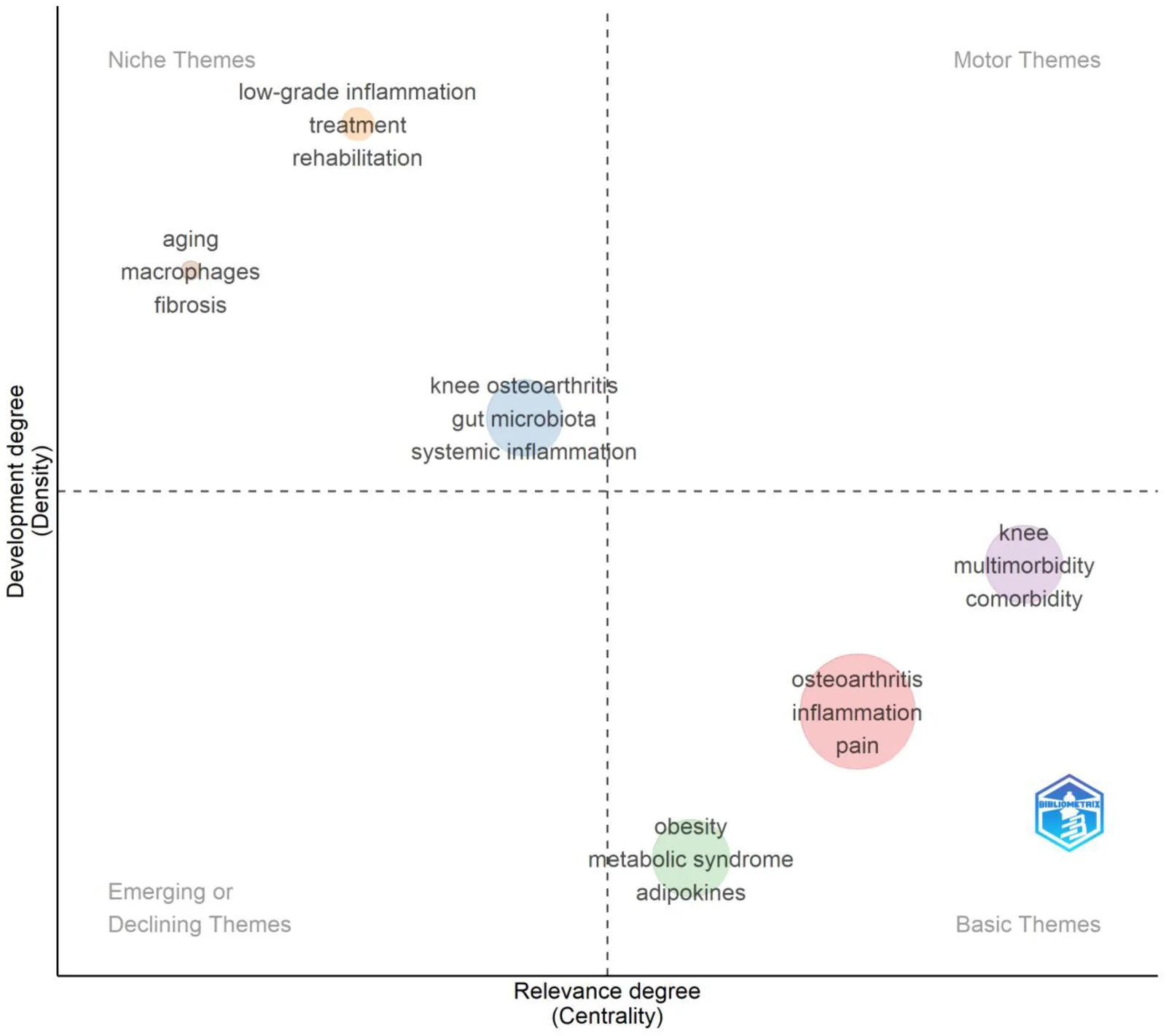
Thematic map based on harmonized Author Keywords. Themes are positioned according to centrality and density, while bubble size represents theme frequency.

The interdisciplinary citation relationships in various research areas, as reflected in dual-map overlay analysis of journals (**Figure 9**), were strong. Clinical medicine journals often cite studies from molecular biology, genetics, and immunology journals, suggesting extensive knowledge sharing between mechanistic basic science and clinical osteoarthritis studies.

**Figure 9:**
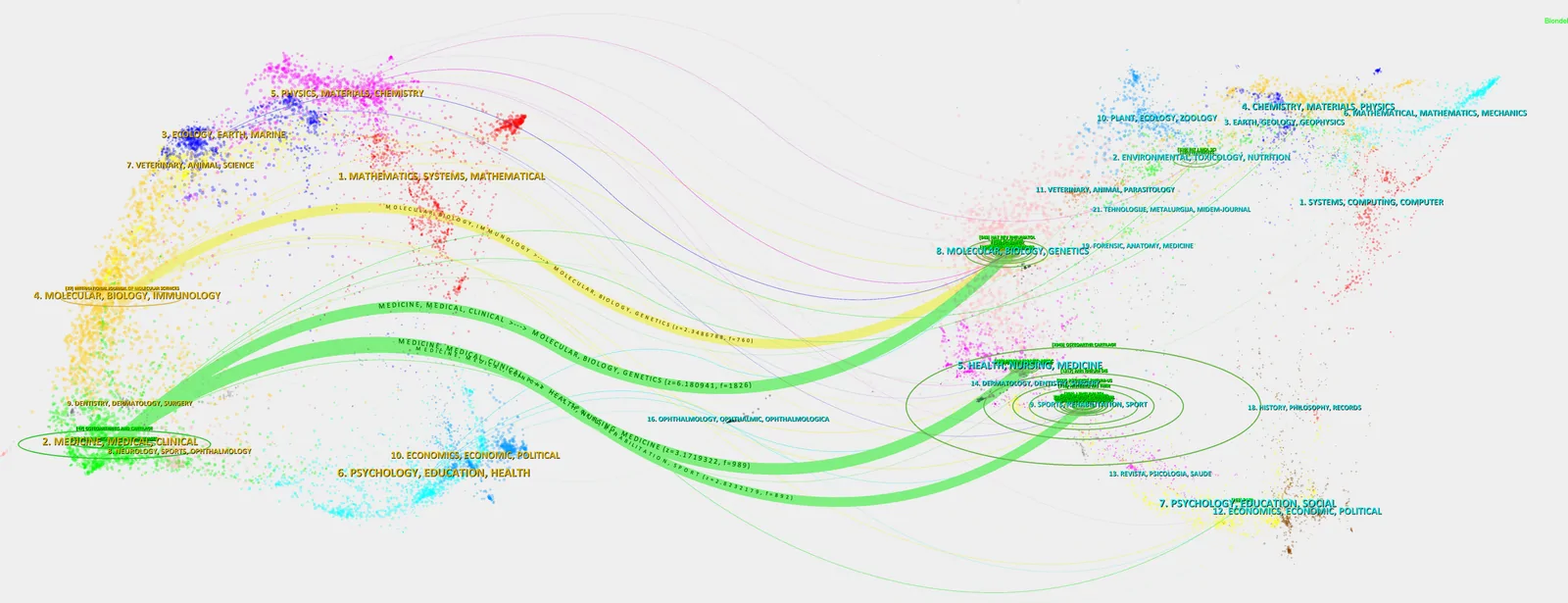
Dual-map overlay of journals illustrating citation pathways.

Overall, the identified research hotspots indicated increasing research attention to multidimensional immunometabolic and translational themes involving metabolic dysfunction, systemic comorbidities, molecular mechanisms, and clinical management strategies.

Sensitivity analyses produced comparable network metrics after excluding 2025 or restricting the dataset to articles. Top-10 keyword overlap with the primary analysis was 80% and 100%, respectively. The Web of Science-only analysis showed 50% overlap and a smaller, more fragmented network, although the leading concepts remained unchanged (**Supplementary Table S2**).

## 4 Discussion

### 4.1 Progressive shift toward immunometabolic and inflammatory perspectives in knee osteoarthritis research

Our bibliometric analysis indicated a progressive shift in research attention from predominantly localized degenerative models toward broader investigation of systemic immunometabolic factors in KOA. Keyword evolution and co-citation analyses showed increasing research emphasis on metabolic dysfunction and chronic low-grade inflammation as potential mechanisms associated with KOA progression (21, 22). The evolving perspective has far-reaching implications for understanding the disease and developing therapeutic strategies.

From a biological perspective, KOA is now known to be a multifactorial phenomenon involving metabolic, inflammatory, and joint-specific processes (21, 23) that lead to cartilage degradation via matrix metalloproteinase activity, reduced extracellular matrix production, and synovial inflammation. Adipose tissue is also an active immunometabolic organ that produces adipokines such as leptin, adiponectin, and resistin, all of which can affect chondrocytes’ metabolism, inflammatory signaling, and subchondral bone turnover (22, 24). Thematic evolution shows growing research attention to innate immune activation, macrophage responses, and NLRP3 signaling; the cited mechanistic literature supports their biological roles (25, 26).

Another mechanism linking metabolic dysfunction to the progressive destruction of joints is oxidative stress. Increased production of ROS in obesity, insulin resistance, and aging also leads to mitochondrial dysfunction, chondrocyte apoptosis, and extracellular matrix degradation (24, 27). Significantly, all these pathways are connected in a feed-forward cycle of metabolic derangements, inflammatory stimulation, oxidative stress, and immune signaling, resulting in greater structural joint degradation (28).

Changes in research emphasis can also be understood through the temporal patterns observed in the present bibliometric study (**Figure 10**). The early studies (2000–2010) emphasized inflammatory changes in the joint microenvironment, whereas later studies emphasized metabolic risk factors, multimorbidity, cardiometabolic dysfunction, and immune-mediated mechanisms (25, 28–30). This progress results from more integrated development of translationally relevant models of immunometabolic diseases.

**Figure 10:**
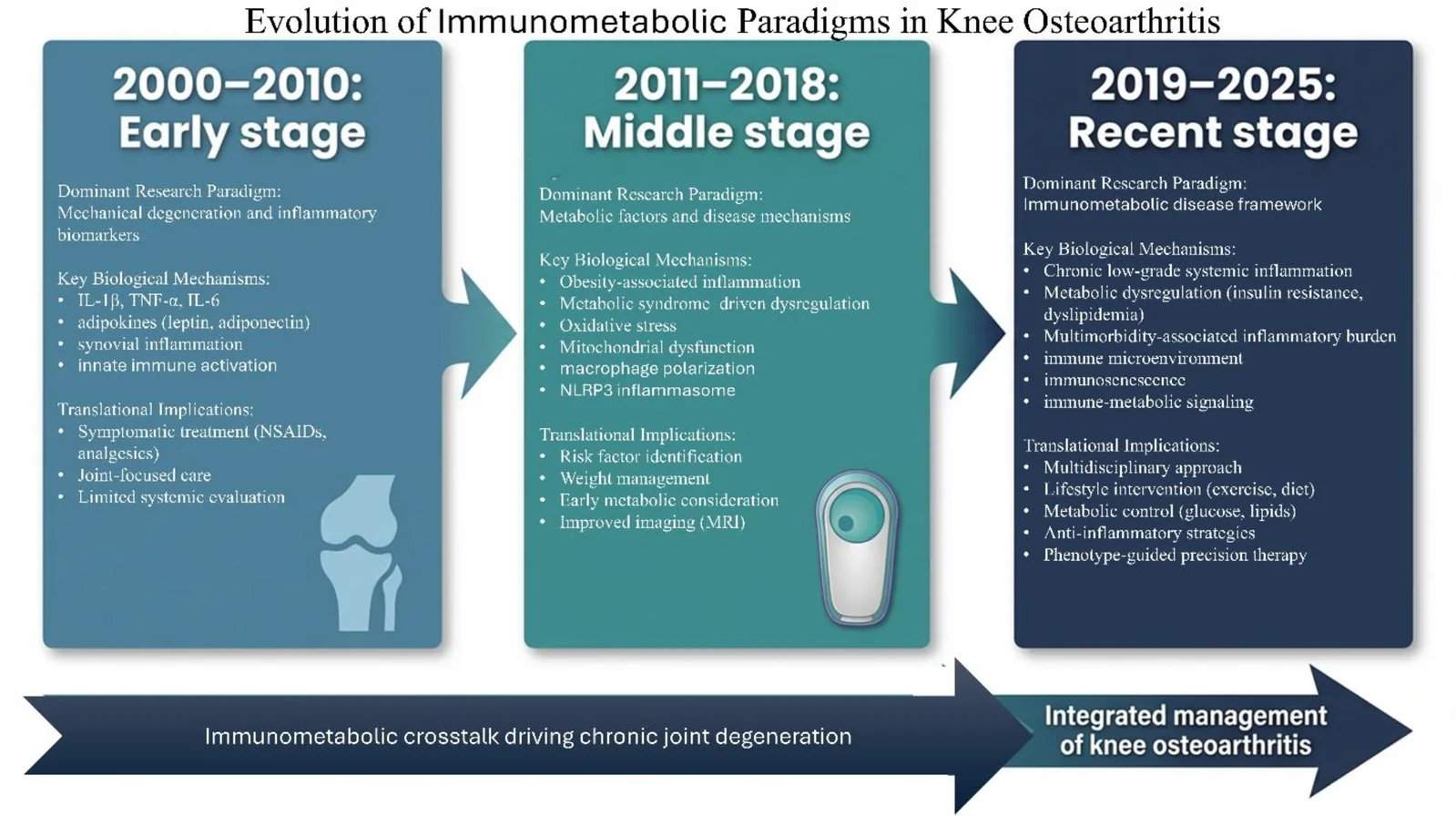
Evolution of research paradigms, underlying biological mechanisms, and translational strategies in knee osteoarthritis (2000–2025).

This evolving framework has clinical implications. The growing investigation of systemic immunometabolic factors may support future research on KOA stratification and personalized care. Interventions targeting metabolic regulation, inflammatory signaling, oxidative stress, and lifestyle modification are increasingly being investigated, but their disease-modifying effects require clinical validation (31–33).

Together, these findings indicate increasing research attention to interactions among metabolic dysfunction, immune activation, inflammatory signaling, and joint degeneration.

### 4.2 Immunometabolic crosstalk as an emerging mechanistic perspective in osteoarthritis pathophysiology

Our keyword co-occurrence and thematic analyses showed increasing research attention toward systemic metabolic dysfunction as a potential contributor to OA pathogenesis. In our bibliometric analysis (**Figure 6B**), we found that metabolic syndrome, chronic inflammation, diabetes mellitus, and multimorbidity were among the main research topics, as was observed for the presence of co-occurring keywords, which has been noted as an increasing trend in the understanding of metabolic influence in the pathogenesis of OA (22, 29). The findings are consistent with the metabolic osteoarthritis hypothesis, in which obesity, dyslipidemia, and insulin resistance contribute to joint deterioration through metabolic and inflammatory pathways (25).

Co-citation and keyword analyses identified adipose tissue–related immunometabolic signaling as a major mechanistic domain in contemporary OA research. Leptin, adiponectin, and resistin are adipokines involved in chondrocyte metabolism, inflammatory signaling, and extracellular matrix degradation (34, 35). These mediators contribute to cartilage destruction by increasing inflammatory cytokine production and activating matrix-degrading enzymes, thereby linking systemic metabolic dysfunction to local joint pathology (35, 36). At the same time, the role of innate immune activation, macrophage-driven inflammatory responses, and NLRP3 inflammasome signaling in sustaining the chronic inflammatory microenvironment of OA joints is gaining increasing traction. Consistent with these findings, co-citation network analysis (**Figure 6A**) identified clusters related to metabolic regulation and chondrocyte activation, emphasizing the growing importance of immunometabolic pathways in contemporary OA research.

In addition to adipokine-mediated mechanisms, chronic, low-grade inflammation and oxidative stress are also implicated in OA progression. It is also known that metabolic syndrome and obesity are associated with increased production of inflammatory cytokines such as interleukin-6 and tumor necrosis factor-α, which are linked to synovial inflammation, cartilage degradation, and subchondral bone remodeling (6, 24, 37). These effects are further potentiated by oxidative stress, which leads to mitochondrial dysfunction, chondrocyte apoptosis, and extracellular matrix degradation, causing a vicious cycle of inflammation and joint destruction (24, 27, 38).

Thematic evolution analysis demonstrated increasing integration between metabolic dysfunction and inflammatory signaling pathways in OA research. The thematic evolution analysis suggests increasing research attention to systemic immunometabolic factors alongside established joint-level perspectives on OA (39, 40). This shifting view has significant translational implications. In addition to traditional joint treatments, therapies targeting metabolic dysfunction, inflammatory signaling, oxidative stress, and lifestyle modification are being explored to identify effective strategies for disease management (33, 41, 42). Together, these discoveries suggest a synergistic mechanistic paradigm in which metabolic dysfunction, immune activation, and inflammatory signaling all contribute to OA development and could inform more targeted treatment strategies.

### 4.3 Emerging research themes and increasing interdisciplinary integration in osteoarthritis

Recent bibliometric trends demonstrated an increasingly adopted, combined, interdisciplinary approach. The burst keyword analysis (**Figure 7**) indicates increased interest in multimorbidity, oxidative stress, lifestyle factors, and immune-related mechanisms, reflecting increasing research attention to systemic factors alongside established joint-level models (43).

Multimorbidity emerged as a major thematic cluster linking OA with cardiometabolic and inflammatory disorders. OA is often comorbid with obesity, diabetes mellitus, hypertension, and cardiovascular disease, and they are interconnected disease networks instead of isolated clinical entities (28, 40, 44). These disorders are characterized by similar mechanisms, including low-grade chronic inflammation, metabolic imbalances, immune stimulation, and age-related biological changes. They are all associated with disease progression and symptom burden. The clustering of these terms in **Figure 6B** reflects increasing research interest in the relationships between OA and cardiometabolic or immunometabolic conditions, without establishing biological causality.

Oxidative stress and inflammatory signaling pathways were consistently identified as expanding mechanistic research hotspots. Excessive production of reactive oxygen species contributes to mitochondrial dysfunction, chondrocyte apoptosis, extracellular matrix degradation, and activation of inflammatory pathways within osteoarthritic tissues (24, 27, 38). The increasing prevalence of oxidative stress-related keywords may indicate a growing interest in the molecular interactions underlying metabolism, inflammation, aging, and immune-mediated tissue remodeling in OA.

Meanwhile, new technologies and translational methodologies are changing the landscape. Advances in molecular profiling, systems biology, and single-cell transcriptomics have enabled improved insights into immune-cell heterogeneity and the synovial inflammatory microenvironment in osteoarthritis. Recent bursts in physical activity, triacylglycerol, and oxidative stress reflect growing interest in lifestyle and metabolic mechanisms. Clinical literature supports weight loss and physical activity for improving symptoms and function, while their broader metabolic and disease-modifying effects remain under investigation (31, 33, 45).

Overall, these findings indicate growing integration across rheumatology, immunology, metabolic medicine, molecular biology, and public health. This interdisciplinary transition is also reflected in the thematic map (**Figure 8**) and journal overlay analysis (**Figure 9**), indicating broader research attention to systems-level perspectives on OA.

### 4.4 Implications for clinical management and future research directions

Our findings support a progressive transition for both clinical practice and future research in osteoarthritis (OA). Growing research on metabolic dysfunction, immune activation, and chronic inflammation reflects increasing attention to systemic factors alongside established joint-level models of OA. Current research increasingly examines OA in relation to cardiometabolic and inflammatory conditions, including obesity, diabetes mellitus, and cardiovascular disease (28, 40, 46).

This changing viewpoint indicates the need for more comprehensive management approaches. In addition to traditional joint-based treatments, weight loss, exercise, and metabolic-risk management are areas of clinical and research interest, but bibliometric patterns cannot infer their disease-modifying effects (31, 33, 45, 47).

Progress in the study of biomarkers, molecular profiling, and immune-related signaling is also contributing to the development of more personalized therapeutic strategies. Inflammatory, metabolic, oxidative-stress, and cartilage-turnover biomarkers are being investigated for potential roles in diagnosis, risk stratification, and treatment selection (48). Additionally, considering individual OA phenotypes that encompass metabolically activated, inflammation-relevant, and biomechanical subtypes could inform more specific, precision-focused interventions.

Future research should focus on integrating molecular mechanisms with clinical, epidemiological, and imaging data to better clarify the relationships among metabolic dysfunction, immune-mediated inflammation, and joint degeneration. New methods, such as systems biology, single-cell transcriptomics, and longitudinal multimodal studies, can further improve disease classification and identify new therapeutic targets (49). Moreover, further research into multimorbidity and cardiometabolic interactions will be necessary to design patient-centered management approaches to the systemic complexity of OA (50).

Together, these results suggest that a more integrated translational model incorporating metabolic dysfunction, immune signaling, and joint pathology should be examined collectively, and that the development of more personalized, mechanism-based interventions to manage OA should be advanced.

### 4.5 Proposed immunometabolic translational framework for knee osteoarthritis

We developed the proposed framework from the recurring themes identified through reference co-citation, keyword co-occurrence and burst analyses, and thematic mapping (**Figures 6**-**8**), interpreted alongside the supporting mechanistic literature. From a systems-biology perspective, these recurring themes connect metabolic risk factors, immune signaling, and joint pathology as interacting research domains rather than isolated mechanisms. The framework is intended as a hypothesis-generating tool for organizing emerging research trends and requires experimental and clinical validation (**Figure 11**).

**Figure 11:**
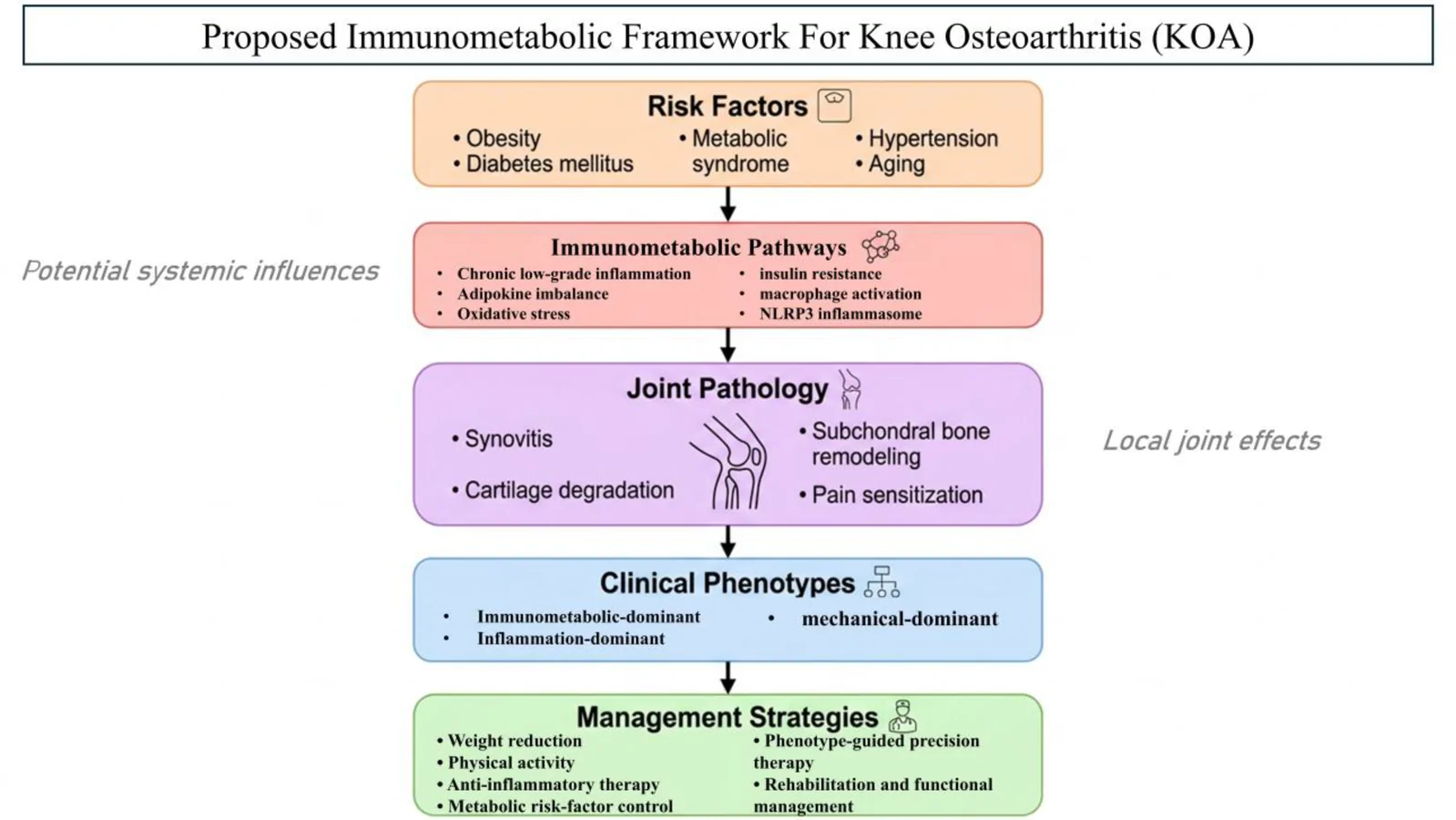
Proposed immunometabolic translational framework for knee osteoarthritis (KOA). This hypothesis-generating framework conceptually organizes proposed relationships among metabolic risk factors, immune-mediated mechanisms, joint pathology, candidate clinical phenotypes, and potential management strategies in KOA. It is informed by the bibliometric, thematic, and mechanistic literature and does not demonstrate causal relationships. Arrows indicate proposed associations rather than established causality. The candidate phenotypes may overlap and require further clinical validation.

Bibliometric and thematic analyses consistently identified obesity, diabetes mellitus, metabolic syndrome, hypertension, and aging as major upstream risk domains (51, 52). These states are strongly linked to metabolic dysregulation and chronic low-grade inflammation and seem to play a role in OA progression through systemic biological processes, not solely through mechanical loading.

Adipokines such as leptin and adiponectin, together with cytokines such as interleukin-6 and tumor necrosis factor-α, are linked to synovial inflammation, cartilage degradation, and extracellular matrix remodeling (53–55). At the same time, oxidative stress and mitochondrial dysfunction contribute to this tissue damage through mechanisms such as chondrocyte apoptosis and inflammatory signaling (56). New evidence also suggests that chronic inflammation in the osteoarthritic joint is maintained by a chronic inflammatory microenvironment, in part via macrophage-mediated inflammation and NLRP3 inflammasome activation.

Systemically, these mechanisms are expressed at the tissue level as synovitis, cartilage degeneration, subchondral bone remodeling, pain progression, and functional deterioration. This synthesized approach places established local joint pathology within a broader hypothesis-generating immunometabolic research perspective (57).

Of particular interest is this framework to stratify patients by phenotype. The framework proposes metabolically dominant, inflammation-dominant, and mechanically dominant candidate phenotypic patterns that may overlap and require clinical validation (58). These proposed patterns may help organize observed variations in disease course and treatment response but require clinical validation.

A translational approach will highlight the value of a holistic management approach when considering metabolic- and immune-mediated pathways. Apart from traditional joint-specific strategies, weight loss, physical exercise, glycemic control, lipid management, and anti-inflammatory therapy are candidate management strategies whose disease-modifying effects require clinical validation (31, 33, 59). Precision-based approaches, including biomarker profiling, molecular phenotyping, and immunometabolic stratification, warrant further evaluation for their potential role in treatment selection and long-term disease management.

Overall, the proposed structure integrates epidemiological trends, biological processes, and translational approaches within a conceptual framework. The model is hypothesis-generating and should not be interpreted as demonstrating causal pathways or validated clinical phenotypes. It also points out possible future lines of research and precision management in KOA.

### 4.6 Strengths and limitations

It has several strengths as a study. First, the inclusion of three of the largest scientific databases in the world–Web of Science, Scopus, and PubMed–helped to make the data set more representative. It decreased the chances of selection bias (60). Second, the study spanned a wide timeframe (2000–2025) to identify temporal trends and changes in priorities, to gain an understanding of the evolution of osteoarthritis research. Third, bibliometric software was used to supplement the analysis of collaboration patterns, intellectual foundations, and research fronts to ensure the validity and interpretation of the results (11, 12).

This study, however, has some shortcomings. First, only English media were examined, so other languages may not have been included, leading to language bias. Second, bibliometric studies depend on the indexing and citation practices of the database used, and the database’s data coverage and normalization may affect the quality of the search results (61). Third, citation analysis might find that older publications are more likely to be cited, and that newer research topics and studies may be under-cited (62).

Finally, descriptive statistics of bibliometric data do not enable causal conclusions about the relationship of research activity to specific biological or clinical mechanisms. Although this study integrates mechanistic interpretation and maximizes its translational potential, it remains a research study rather than a study of disease mechanisms. These findings will have to be confirmed and expanded with experimental, clinical, and longitudinal studies.

In conclusion, the present study has some limitations. Still, it offers a broad, sound, and dynamic overview of the complex field of research on knee OA and its mechanisms, highlighting the potential for translation into research and practice.

## 5 Conclusion

This bibliometric study provides an overview of recent developments in the research area of the relationship between knee osteoarthritis (KOA) and immunometabolic and inflammatory processes during the 2000–2025 study period. The results show increasing research attention to systems-level factors—including metabolic dysfunction, chronic low-grade inflammation, immune activation, and lifestyle-related factors—alongside established joint-centered approaches.

Furthermore, the review reflects the growing interdisciplinarity in contemporary KOA research, with increasing interest in immunometabolic signaling, multimorbidity, molecular pathways, and translational medicine. These trends reflect increasing investigation of KOA in relation to cardiometabolic and immune-related conditions.

The developing knowledge can serve as the foundation for more personalized, mechanism-based management. As with traditional biomechanical pathways, metabolic, inflammatory, oxidative stress, and immune-mediated pathways are important and may provide additional strategies for better disease stratification, earlier intervention, and better long-term clinical outcomes. Additionally, increasing awareness of the phenotypic patterns of a disease can aid in the possibility of precision treatment for KOA.

In this paper, we have included both bibliometric and mechanistic evidence, along with a translation interpretation, to present the new trends in KOA research systematically. The proposed framework may be useful for future investigations and for designing more comprehensive and targeted knee OA prevention and treatment strategies that encompass epidemiological dynamics, immunometabolic responses, and clinical implications.

## Data Availability

The original contributions presented in the study are included in the article/**Supplementary Material**. Further inquiries can be directed to the corresponding author.

## References

[1] Katz JN Arant KR Loeser RF . Diagnosis and treatment of hip and knee osteoarthritis: a review. JAMA. (2021) 325:568–78. doi: 10.1001/jama.2020.22171 33560326 PMC8225295

[2] Safiri S Kolahi AA Smith E Hill C Bettampadi D Mansournia MA . Global, regional and national burden of osteoarthritis 1990–2017: a systematic analysis of the Global Burden of Disease Study 2017. Ann Rheum Dis. (2020) 79:819–28. doi: 10.1136/annrheumdis-2019-216515 32398285

[3] Cross M Smith E Hoy D Nolte S Ackerman I Fransen M . The global burden of hip and knee osteoarthritis. Ann Rheum Dis. (2014) 73:1323–30. doi: 10.1136/annrheumdis-2013-204763 24553908

[4] Vos T Lim SS Abbafati C Abbas KM Abbasi M Abbasifard M . Global burden of 369 diseases and injuries in 204 countries and territories, 1990–2019: a systematic analysis for the Global Burden of Disease Study 2019. Lancet. (2020) 396:1204–22. doi: 10.1016/S0140-6736(20)30925-9 33069326 PMC7567026

[5] Goldring MB Goldring SR . Articular cartilage and subchondral bone in the pathogenesis of osteoarthritis. Ann N Y Acad Sci. (2010) 1192:230–7. doi: 10.1111/j.1749-6632.2009.05240.x 20392241

[6] Robinson WH Lepus CM Wang Q Raghu H Mao R Lindstrom TM . Low-grade inflammation as a key mediator of the pathogenesis of osteoarthritis. Nat Rev Rheumatol. (2016) 12:580–92. doi: 10.1038/nrrheum.2016.136 27539668 PMC5500215

[7] Berenbaum F . Osteoarthritis as an inflammatory disease (osteoarthritis is not osteoarthrosis!). Osteoarthritis Cartilage. (2013) 21:16–21. doi: 10.1016/j.joca.2012.11.012 23194896

[8] Neogi T . The epidemiology and impact of pain in osteoarthritis. Osteoarthritis Cartilage. (2013) 21:1145–53. doi: 10.1016/j.joca.2013.03.018 23973124 PMC3753584

[9] Bijlsma JWJ Berenbaum F Lafeber FPJG . Osteoarthritis: an update with relevance for clinical practice. Lancet. (2011) 377:2115–26. doi: 10.1016/S0140-6736(11)60243-2 21684382

[10] Grant MJ Booth A . A typology of reviews: an analysis of 14 review types and associated methodologies. Health Info Libr J. (2009) 26:91–108. doi: 10.1111/j.1471-1842.2009.00848.x 19490148

[11] Donthu N Kumar S Mukherjee D Pandey N Lim WM . How to conduct a bibliometric analysis: an overview and guidelines. J Bus Res. (2021) 133:285–96. doi: 10.1016/j.jbusres.2021.04.07042574925

[12] Ellegaard O Wallin JA . The bibliometric analysis of scholarly production: How great is the impact? Scientometrics. (2015) 105:1809–31. doi: 10.1007/s11192-015-1645-z 26594073 PMC4643120

[13] Chen C . CiteSpace II: Detecting and visualizing emerging trends and transient patterns in scientific literature. J Am Soc Inf Sci Technol. (2006) 57:359–77. doi: 10.1002/asi.2031742587375

[14] Aria M Cuccurullo C . Bibliometrix: An R-tool for comprehensive science mapping analysis. J Informetr. (2017) 11:959–75. doi: 10.1016/j.joi.2017.08.007

[15] van Eck NJ Waltman L . Software survey: VOSviewer, a computer program for bibliometric mapping. Scientometrics. (2010) 84:523–38. doi: 10.1007/s11192-009-0146-3 20585380 PMC2883932

[16] Small H . Co-citation in the scientific literature: A new measure of the relationship between two documents. J Am Soc Inf Sci. (1973) 24:265–9. doi: 10.1002/asi.463024040642587375

[17] Falagas ME Pitsouni EI Malietzis GA Pappas G . Comparison of pubMed, scopus, web of science. FASEB J. (2008) 22:338–42. doi: 10.1096/fj.07-9492LSF 17884971

[18] Chen C . Citespace: A Practical Guide for Mapping Scientific Literature. New York: Nova Science Publishers (2016).

[19] Callon M Courtial JP Laville F . Co-word analysis as a tool for describing the network of interactions between basic and technological research. Scientometrics. (1991) 22:155–205. doi: 10.1007/bf0201928030311153

[20] Moher D Liberati A Tetzlaff J Altman DG . Preferred reporting items for systematic reviews and meta-analyses: The PRISMA statement. PloS Med. (2009) 6:e1000097. doi: 10.1371/journal.pmed.1000097 PMC2707599 19621072

[21] Kapoor M Martel-Pelletier J Lajeunesse D Pelletier JP Fahmi H . Role of proinflammatory cytokines in the pathophysiology of osteoarthritis. Nat Rev Rheumatol. (2011) 7:33–42. doi: 10.1038/nrrheum.2010.196 21119608

[22] Mobasheri A Rayman MP Gualillo O Sellam J van der Kraan P Fearon U . The role of metabolism in the pathogenesis of osteoarthritis. Nat Rev Rheumatol. (2017) 13:302–11. doi: 10.1038/nrrheum.2017.50 28381830

[23] Scanzello CR Goldring SR . The role of synovitis in osteoarthritis pathogenesis. Bone. (2012) 51:249–57. doi: 10.1016/j.bone.2012.02.012 22387238 PMC3372675

[24] Lepetsos P Papavassiliou AG . ROS/oxidative stress signaling in osteoarthritis. Biochim Biophys Acta. (2016) 1862:576–91. doi: 10.1016/j.bbadis.2016.01.003 26769361

[25] Puenpatom RA Victor TW . Increased prevalence of metabolic syndrome in individuals with osteoarthritis: An analysis of NHANES III data. Postgrad Med. (2009) 121:9–20. doi: 10.3810/pgm.2009.11.2073 19940413

[26] Loeser RF Goldring SR Scanzello CR Goldring MB . Osteoarthritis: A disease of the joint as an organ. Arthritis Rheum. (2012) 64:1697–707. doi: 10.1002/art.34453 22392533 PMC3366018

[27] Henrotin Y Kurz B Aigner T . Oxygen and reactive oxygen species in cartilage degradation: Friends or foes? Osteoarthritis Cartilage. (2005) 13:643–54. doi: 10.1016/j.joca.2005.04.002 15936958

[28] Herrero-Beaumont G Roman-Blas JA Castañeda S Jiménez SA . Primary osteoarthritis no longer primary: three subsets with distinct etiological, clinical, and therapeutic characteristics. Semin Arthritis Rheum. (2009) 39:71–80. doi: 10.1016/j.semarthrit.2008.04.004 19589561

[29] Kraus VB Blanco FJ Englund M Karsdal MA Lohmander LS . Call for standardized definitions of osteoarthritis and risk stratification. Osteoarthritis Cartilage. (2015) 23:1233–41. doi: 10.1016/j.joca.2015.03.036 25865392 PMC4516635

[30] Kadam UT Jordan KP Croft PR . Clinical comorbidity in patients with osteoarthritis. Ann Rheum Dis. (2004) 63:408–14. doi: 10.1136/ard.2003.009548 15020335 PMC1754944

[31] Bannuru RR Osani MC Vaysbrot EE Arden NK Bennell K Bierma-Zeinstra SMA . OARSI guidelines for the non-surgical management of knee, hip, and polyarticular osteoarthritis. Osteoarthritis Cartilage. (2019) 27:1578–89. doi: 10.1016/j.joca.2019.06.011 31278997

[32] Messier SP Mihalko SL Legault C Miller GD Nicklas BJ DeVita P . Effects of intensive diet and exercise on knee osteoarthritis. JAMA. (2013) 310:1263–73. doi: 10.1001/jama.2013.277669 24065013 PMC4450354

[33] Fransen M McConnell S Harmer AR Van der Esch M Simic M Bennell KL . Exercise for osteoarthritis of the knee. Cochrane Database Syst Rev. (2015) 2015(1):CD004376. doi: 10.1002/14651858.CD004376.pub3 25569281 PMC10094004

[34] Ouchi N Parker JL Lugus JJ Walsh K . Adipokines in inflammation and metabolic disease. Nat Rev Immunol. (2011) 11:85–97. doi: 10.1038/nri2921 21252989 PMC3518031

[35] Conde J Scotece M Gómez R López V Gómez-Reino JJ Gualillo O . Adipokines and osteoarthritis: novel molecules involved in the pathogenesis and progression of disease. Arthritis. (2011) 2011:203901. doi: 10.1155/2011/203901 22046513 PMC3200120

[36] Lago F Gómez R Gómez-Reino JJ Dieguez C Gualillo O . Adipokines as emerging mediators of immune response and inflammation. Nat Clin Pract Rheumatol. (2007) 3:716–24. doi: 10.1038/ncprheum0674 18037931

[37] Wang T He C . Pro-inflammatory cytokines: The link between obesity and osteoarthritis. Cytokine Growth Factor Rev. (2018) 44:38–50. doi: 10.1016/j.cytogfr.2018.10.002 30340925

[38] Ziskoven C Jäger M Zilkens C Bloch W Brixius K Krauspe R . Oxidative stress in secondary osteoarthritis: from cartilagedestruction to clinical presentation? Orthop Rev (Pavia). (2010) 2. doi: 10.4081/or.2010.e23 PMC3143971 21808712

[39] Louati K Vidal C Berenbaum F Sellam J . Association between diabetes mellitus and osteoarthritis. RMD Open. (2015) 1:e000077. doi: 10.1136/rmdopen-2015-000077 26535137 PMC4613158

[40] Swain S Sarmanova A Coupland C Doherty M Zhang W . Comorbidities in osteoarthritis. Ann Rheum Dis. (2020) 79:681–8. doi: 10.1136/annrheumdis-2019-216374 32079570 PMC7147174

[41] Messier SP . Diet and exercise for obese adults with knee osteoarthritis. Clin Geriatr Med. (2010) 26:461–77. doi: 10.1016/j.cger.2010.05.001 20699166 PMC3444812

[42] Hunter DJ Bierma-Zeinstra S . Osteoarthritis. Lancet. (2019) 393:1745–59. doi: 10.1016/S0140-6736(19)30417-9 31034380

[43] Briggs AM Woolf AD Dreinhöfer K Homb N Hoy DG Kopansky-Giles D . Reducing the global burden of musculoskeletal conditions. Best Pract Res Clin Rheumatol. (2018) 32:221–33. doi: 10.1016/j.berh.2018.08.002 29875522 PMC5985424

[44] Veronese N Cereda E Maggi S Luchini C Solmi M Smith T . Osteoarthritis and mortality: A systematic review and meta-analysis. Semin Arthritis Rheum. (2016) 46:160–7. doi: 10.1016/j.semarthrit.2016.04.002 27179749

[45] Skou ST Roos EM . Good Life with osteoArthritis in Denmark (GLA:D™): evidence-based education and supervised neuromuscular exercise delivered by certified physiotherapists nationwide. BMC Musculoskelet Disord. (2017) 18:72. doi: 10.1186/s12891-017-1439-y PMC5297181 28173795

[46] Scanzello CR . Role of low-grade inflammation in osteoarthritis. Curr Opin Rheumatol. (2017) 29:79–85. doi: 10.1097/BOR.0000000000000353 27755180 PMC5565735

[47] Vincent HK Heywood K Connelly J Hurley RW . Obesity and weight loss in the treatment and prevention of osteoarthritis. PM&R. (2012) 4:S59–67. doi: 10.1016/j.pmrj.2012.01.005 22632704 PMC3623013

[48] Kraus VB . Biomarkers in osteoarthritis. Curr Opin Rheumatol. (2018) 30:101–7. doi: 10.1097/BOR.0000000000000452 16093846

[49] Loeser RF . Aging and osteoarthritis. Curr Opin Rheumatol. (2011) 23:492–6. doi: 10.1097/BOR.0b013e3283494005 21709557 PMC3377970

[50] Felson DT . Osteoarthritis as a disease of mechanics. Osteoarthritis Cartilage. (2013) 21:10–5. doi: 10.1016/j.joca.2012.09.012 23041436 PMC3538894

[51] Zheng H Chen C . Body mass index and risk of knee osteoarthritis: systematic review and meta-analysis. Am J Epidemiol. (2015) 181:707–17. doi: 10.1093/aje/kwu309 26656979 PMC4679914

[52] Courties A Sellam J Berenbaum F . Metabolic syndrome-associated osteoarthritis. Curr Opin Rheumatol. (2017) 29:214–22. doi: 10.1097/BOR.0000000000000373 28072592

[53] Sokolove J Lepus CM . Role of inflammation in the pathogenesis of osteoarthritis: latest findings and interpretations. Ther Adv Musculoskelet Dis. (2013) 5:77–94. doi: 10.1177/1759720X12467868 23641259 PMC3638313

[54] Goldring SR Goldring MB . Clinical aspects, pathology and pathophysiology of osteoarthritis. J Musculoskelet Neuronal Interact. (2006) 6:376–8. 17185832

[55] Xie C Chen Q . Adipokines: new therapeutic target for osteoarthritis? Curr Rheumatol Rep. (2019) 21:71. doi: 10.1007/s11926-019-0868-z 31813080 PMC7291783

[56] Bolduc JA Collins JA Loeser RF . Reactive oxygen species, aging and articular cartilage homeostasis. Free Radic Biol Med. (2019) 132:73–82. doi: 10.1016/j.freeradbiomed.2018.08.038 30176344 PMC6342625

[57] Loeser RF . The role of aging in the development of osteoarthritis. Trans Am Clin Climatol Assoc. (2017) 128:44–54. PMC5525396 28790486

[58] Deveza LA Melo L Yamato TP Mills K Ravi V Hunter DJ . Knee osteoarthritis phenotypes and their relevance for outcomes: a systematic review. Osteoarthritis Cartilage. (2017) 25:1926–41. doi: 10.1016/j.joca.2017.08.009 28847624

[59] Kolasinski SL Neogi T Hochberg MC Oatis C Guyatt G Block J . 2019 American College of Rheumatology/Arthritis Foundation guideline for the management of osteoarthritis of the hand, hip, and knee. Arthritis Care Res (Hoboken). (2020) 72:149–62. doi: 10.1002/acr.24131 31908149 PMC11488261

[60] Bramer WM Rethlefsen ML Kleijnen J Franco OH . Optimal database combinations for literature searches in systematic reviews: a prospective exploratory study. Syst Rev. (2017) 6:245. doi: 10.1186/s13643-017-0644-y 29208034 PMC5718002

[61] Mongeon P Paul-Hus A . The journal coverage of Web of Science and Scopus. Scientometrics. (2016) 106:213–28. doi: 10.1007/s11192-015-1765-530311153

[62] Tahamtan I Safipour Afshar A Ahamdzadeh K . Factors affecting citation rates of scientific articles. Scientometrics. (2016) 107:1195–225. doi: 10.1007/s11192-016-1889-230311153

