# Supplementary material for: Immunometabolic crosstalk in knee osteoarthritis: a bibliometric and translational analysis (2000–2025)
Source: Front Immunol. 2026 Sep 17;17:1877104. doi: 10.3389/fimmu.2026.1877104 (PMC13627379; doi:10.3389/fimmu.2026.1877104)
Supplement: Supplementary file 1 [file SupplementaryFile1.docx]

Supplementary File S1. Detailed Search Strategies for Each Database

# General Information

- Databases searched: Web of Science Core Collection, Scopus, PubMed
- Search date: 15 February 2026
- Time span: 1 January 2000 – 31 December 2025
- Language restriction: English
- Document types: Articles and Reviews

# Scopus Search Strategy

TITLE-ABS-KEY (
("knee osteoarthritis" OR "knee OA" OR gonarthrosis OR gonarth*
 OR "tibiofemoral osteoarthritis" OR "patellofemoral osteoarthritis")
AND
(
"metabolic osteoarthritis"
OR "metabolic phenotype"
OR "systemic inflammation"
OR "low-grade inflammation"
OR "metabolic disorder*"
OR "chronic systemic disease*"
OR "multimorbidity"
OR "cardiometabolic"
))
AND (PUBYEAR > 1999 AND PUBYEAR < 2026)
AND (LIMIT-TO (DOCTYPE, "ar") OR LIMIT-TO (DOCTYPE, "re"))
AND (LIMIT-TO (LANGUAGE, "English"))

Results:

- Initial records: 430
- After filtering: 403
- Articles: 326
- Reviews: 77

# Web of Science Search Strategy

TS = (
("knee osteoarthritis" OR "knee OA" OR gonarthrosis OR gonarth*
 OR "tibiofemoral osteoarthritis" OR "patellofemoral osteoarthritis")
AND
("metabolic osteoarthritis"
 OR "metabolic phenotype"
 OR "systemic inflammation"
 OR "low-grade inflammation"
 OR "metabolic disorder*"
 OR "chronic systemic disease*"
 OR multimorbidity
 OR cardiometabolic)
)
Timespan: 2000–2025
Indexes: SCI-EXPANDED
Document types: Article, Review
Language: English

Results:

- Initial records: 423
- After filtering: 402
- Articles: 280
- Reviews: 122

# PubMed Search Strategy

(("Osteoarthritis, Knee"[Mesh]
 OR "knee osteoarthritis"[Title/Abstract]
 OR "knee OA"[Title/Abstract]
 OR gonarthrosis[Title/Abstract])
AND
("Metabolic Syndrome"[Mesh]
 OR "Inflammation"[Mesh]
 OR "metabolic osteoarthritis"[Title/Abstract]
 OR "metabolic phenotype"[Title/Abstract]
 OR "systemic inflammation"[Title/Abstract]
 OR "low-grade inflammation"[Title/Abstract]
 OR cardiometabolic[Title/Abstract]
 OR multimorbidity[Title/Abstract]))AND english[Language] AND (journal article[Publication Type] OR review[Publication Type])
AND ("2000/01/01"[Date - Publication] : "2025/12/31"[Date - Publication])

Results:

- Initial records: 722
- After filtering: 422

# Final Dataset Summary

- Total records before filtering: 1575
- After applying filters: 1227
- After deduplication and eligibility assessment: 783 records included in the final analysis.

Of the 1,227 filtered records, we removed 444 duplicate or ineligible records during dataset merging and eligibility assessment. The substantial overlap primarily resulted from the same publications being indexed across Web of Science, Scopus, and PubMed. Duplicate records were identified using DOI, title, author, and publication-year information.
