# Supplementary material for: Immunometabolic crosstalk in knee osteoarthritis: a bibliometric and translational analysis (2000–2025)
Source: Front Immunol. 2026 Sep 17;17:1877104. doi: 10.3389/fimmu.2026.1877104 (PMC13627379; doi:10.3389/fimmu.2026.1877104)
Supplement: Supplementary file 2 [file SupplementaryFile2.docx]

**Supplementary File S2. Data Processing and Bibliometric Analysis Parameters**

This file reports the data-processing, counting, normalization, threshold, and network-construction settings used for the bibliometric analyses.

# 1. Dataset and preprocessing

| **Parameter** | **Setting used** |
| --- | --- |
| **Databases** | Web of Science Core Collection (SCI-EXPANDED), Scopus, and PubMed |
| **Search date** | 15 February 2026 |
| **Publication period** | 1 January 2000 to 31 December 2025 |
| **Eligibility filters** | English-language journal articles and reviews |
| **Initial file formats** | Web of Science: plain text, full records and cited references; Scopus: CSV; PubMed: NBIB |
| **Format harmonization** | We converted Scopus and PubMed exports to CiteSpace-compatible plain-text records before merging. |
| **Deduplication** | CiteSpace built-in duplicate removal based on core bibliographic fields; residual duplicates and invalid records were checked during title and abstract screening. |
| **Keyword preprocessing** | Synonymous variants were merged and indexing artifacts or nonspecific measurement terms were excluded using the rules listed in Table S1. |
| **Final corpus** | 783 unique eligible records |

# 2. CiteSpace settings

| **Parameter** | **Setting used** |
| --- | --- |
| **Software** | CiteSpace 6.4.R1 (64-bit) Advanced |
| **Time slicing** | 2000-2025; 1 year per slice |
| **Term sources** | Title, Abstract, Author Keywords (DE), and Keywords Plus/index terms (ID), as required by the analysis |
| **Node selection** | g-index, k = 25 |
| **Link settings** | Cosine link strength; link-retaining factor = 2.5; links per node = 10; look-back years = 5; e = 1.0 |
| **Pruning** | Pathfinder and pruning sliced networks |
| **Node types analyzed** | Institutions, authors, journals, cited references, and keywords |
| **Reference co-citation** | Cluster view; reported network size, links, density, modularity Q, and mean silhouette. |
| **Keyword co-occurrence** | Harmonized keyword fields; cluster and timeline visualizations |
| **Keyword burst detection** | Harmonized Author Keywords and Keywords Plus/index terms; 20 strongest bursts displayed |

# 3. VOSviewer settings

| **Parameter** | **Setting used** |
| --- | --- |
| **Software** | VOSviewer 1.6.20 |
| **Analysis** | Country co-authorship/collaboration network |
| **Unit of analysis** | Countries |
| **Counting method** | Full counting |
| **Inclusion threshold** | Minimum of 5 publications per country |
| **Normalization** | Association-strength normalization |
| **Clustering and layout** | VOSviewer 1.6.20 default clustering and layout settings |
| **Visualization** | Network visualization; node size represents publication output, link thickness represents collaboration strength, and colors identify clusters |

# 4. Bibliometrix/Biblioshiny settings

| **Parameter** | **Setting used** |
| --- | --- |
| **Software** | Bibliometrix R package 4.1.4, using the Biblioshiny interface |
| **Descriptive analyses** | Annual production, citation indicators, leading authors and sources, Bradford's Law, and geographic production |
| **Thematic-map field** | Harmonized Author Keywords (DE) |
| **Number of words** | 198 |
| **Minimum cluster frequency** | 5 per 1,000 documents |
| **Alpha parameter** | 0.5 |
| **Label display** | Avoid label overlap enabled |
| **Other options** | Biblioshiny 4.1.4 default normalization and clustering options were retained unless specified above |

# 5. Analysis-specific data availability

All three databases contributed core publication metadata. Web of Science and Scopus supplied citation counts and cited-reference data for citation-dependent analyses, whereas PubMed additionally contributed PMID and MeSH data. We incorporated records into each analysis based on the metadata required by the relevant software function; records lacking cited-reference data did not contribute to reference co-citation networks.

# 6. Interpretation of network metrics

| **Parameter** | **Setting used** |
| --- | --- |
| **Node size** | Publication, occurrence, citation, or co-citation frequency, as stated in the corresponding figure legend |
| **Link** | Collaboration, co-occurrence, or co-citation relationship, depending on node type |
| **Link thickness** | Strength of the corresponding relationship |
| **Color** | Cluster membership or publication time, as stated in the corresponding figure legend |
| **Betweenness centrality** | Structural bridging position within a network; it was not interpreted as methodological quality or clinical importance |
| **Modularity Q** | Separation of a network into clusters |
| **Mean silhouette** | Internal consistency of the identified clusters |
