# Supplementary material for: Immunometabolic crosstalk in knee osteoarthritis: a bibliometric and translational analysis (2000–2025)
Source: Front Immunol. 2026 Sep 17;17:1877104. doi: 10.3389/fimmu.2026.1877104 (PMC13627379; doi:10.3389/fimmu.2026.1877104)
Supplement: Supplementary file 3 [file SupplementaryFile3.docx]

**Supplementary File S3. PRISMA 2020 Checklist**

*Adapted reporting checklist for a bibliometric and scientometric analysis*

**Scope note.** This study is not a PICOS-based systematic review. PRISMA 2020 was used to improve transparency in literature identification, deduplication, screening and inclusion. Items specific to intervention-effect synthesis, meta-analysis, risk-of-bias assessment or certainty grading are marked not applicable (N/A).

| **Section** | **Topic** | **Item** | **PRISMA 2020 checklist item** | **Location where reported** |
| --- | --- | --- | --- | --- |
| **TITLE** | Title | **1** | Identify the report as a systematic review. | N/A — the report is identified as a bibliometric and scientometric analysis, not a systematic review. |
| **ABSTRACT** | Abstract | **2** | See the PRISMA 2020 for Abstracts checklist. | Abstract — structured summary of the bibliometric study. |
| **INTRODUCTION** | Rationale | **3** | Describe the rationale for the review in the context of existing knowledge. | Introduction — background and rationale. |
|  | Objectives | **4** | Provide an explicit statement of the objective(s) or question(s) the review addresses. | Introduction — final paragraph. |
| **METHODS** | Eligibility criteria | **5** | Specify the inclusion and exclusion criteria and how studies were grouped for syntheses. | Methods, Section 2.2 (Eligibility Criteria and Study Selection). |
|  | Information sources | **6** | Specify all databases, registers, websites, organisations, reference lists and other sources searched; state the date each source was last searched. | Methods, Section 2.1; Supplementary File S1. |
|  | Search strategy | **7** | Present the full search strategies for all databases, registers and websites, including filters and limits used. | Supplementary File S1. |
|  | Selection process | **8** | Specify the methods used to decide whether a record met the inclusion criteria, including reviewers, independence and automation tools, if applicable. | Methods, Section 2.2. |
|  | Data collection process | **9** | Specify the methods used to collect data from reports, including reviewers, independence, author contact and automation tools, if applicable. | Methods, Sections 2.2–2.3; adapted to bibliographic metadata collection. |
|  | Data items — outcomes | **10a** | List and define all outcomes for which data were sought. | N/A — no clinical outcome synthesis was conducted. |
|  | Data items — other variables | **10b** | List and define all other variables for which data were sought and describe assumptions about missing or unclear information. | Methods, Section 2.3 — bibliometric variables and network indicators. |
|  | Study risk of bias assessment | **11** | Specify the methods used to assess risk of bias in included studies. | N/A — individual-study risk of bias was not assessed in this bibliometric analysis. |
|  | Effect measures | **12** | Specify the effect measure(s) used for each outcome. | N/A — no intervention effects or meta-analysis were evaluated. |
|  | Synthesis methods | **13a** | Describe the processes used to decide which studies were eligible for each synthesis. | Methods, Section 2.2. |
|  |  | **13b** | Describe methods required to prepare data for presentation or synthesis. | Methods, Sections 2.2–2.3 — deduplication, cleaning and keyword harmonisation. |
|  |  | **13c** | Describe methods used to tabulate or visually display results. | Methods, Section 2.3. |
|  |  | **13d** | Describe methods used to synthesize results and justify the choices; describe meta-analysis methods if used. | Methods, Section 2.3 — bibliometric and scientometric analyses; no meta-analysis. |
|  |  | **13e** | Describe methods used to explore possible causes of heterogeneity. | N/A — no quantitative effect synthesis was conducted. |
|  |  | **13f** | Describe sensitivity analyses conducted to assess robustness. | Methods — sensitivity-analysis paragraph. |
|  | Reporting bias assessment | **14** | Describe methods used to assess risk of bias due to missing results in a synthesis. | N/A — no outcome synthesis was conducted. |
|  | Certainty assessment | **15** | Describe methods used to assess certainty in the body of evidence. | N/A — certainty grading was outside the scope of the bibliometric analysis. |
| **RESULTS** | Study selection | **16a** | Describe the search and selection results from records identified to studies included, ideally using a flow diagram. | Results, Section 3.1; Figure 1. |
|  |  | **16b** | Cite studies that appeared eligible but were excluded and explain why. | N/A — exclusions were reported at record level; this was not an intervention review. |
|  | Study characteristics | **17** | Cite each included study and present its characteristics. | N/A — the unit of analysis was the aggregate bibliographic dataset. |
|  | Risk of bias in studies | **18** | Present risk-of-bias assessments for each included study. | N/A. |
|  | Results of individual studies | **19** | Present summary statistics and effect estimates for each study. | N/A — no clinical effect synthesis was performed. |
|  | Results of syntheses | **20a** | Summarise the characteristics and risk of bias among studies contributing to each synthesis. | N/A. |
|  |  | **20b** | Present results of all statistical syntheses, including summary estimates, precision and heterogeneity where applicable. | N/A — bibliometric indicators and networks are reported in Results, Sections 3.1–3.8. |
|  |  | **20c** | Present investigations of possible causes of heterogeneity. | N/A. |
|  |  | **20d** | Present results of all sensitivity analyses. | Methods — sensitivity-analysis paragraph and reported stability of core findings. |
|  | Reporting biases | **21** | Present assessments of risk of bias due to missing results for each synthesis. | N/A. |
|  | Certainty of evidence | **22** | Present assessments of certainty in the body of evidence. | N/A. |
| **DISCUSSION** | Discussion | **23a** | Provide a general interpretation of the results in the context of other evidence. | Discussion, Sections 4.1–4.5. |
|  |  | **23b** | Discuss limitations of the evidence included in the review. | Discussion, Strengths and Limitations. |
|  |  | **23c** | Discuss limitations of the review processes used. | Discussion, Strengths and Limitations. |
|  |  | **23d** | Discuss implications for practice, policy and future research. | Discussion, Sections 4.4–4.5; Conclusion. |
| **OTHER INFORMATION** | Registration and protocol | **24a** | Provide registration information or state that the review was not registered. | Not registered; the study was a bibliometric analysis rather than a systematic review. |
|  |  | **24b** | Indicate where the protocol can be accessed or state that a protocol was not prepared. | No prospective systematic-review protocol was prepared. |
|  |  | **24c** | Describe and explain amendments to registration or protocol information. | N/A. |
|  | Support | **25** | Describe sources of financial or non-financial support and the role of funders or sponsors. | Funding statement. |
|  | Competing interests | **26** | Declare competing interests of the authors. | Conflict of Interest statement. |
|  | Availability of data, code and other materials | **27** | Report which data, code and other materials are publicly available and where they can be found. | Data Availability Statement; supplementary files. |

**Source:** Page MJ, McKenzie JE, Bossuyt PM, et al. The PRISMA 2020 statement: an updated guideline for reporting systematic reviews. BMJ. 2021;372:n71. doi:10.1136/bmj.n71. Checklist adapted under CC BY 4.0.
