# Supplementary material for: Immunometabolic crosstalk in knee osteoarthritis: a bibliometric and translational analysis (2000–2025)
Source: Front Immunol. 2026 Sep 17;17:1877104. doi: 10.3389/fimmu.2026.1877104 (PMC13627379; doi:10.3389/fimmu.2026.1877104)
Supplement: Supplementary file 4 [file Table1.docx]

**Supplementary Table S1. Keyword Harmonization Protocol**

**Related analysis:** Keyword burst analysis (Figure 7) and thematic mapping (Figure 8)

# S1.1 Harmonization procedure

Keyword harmonization was restricted to the Author Keywords (DE) and Keywords Plus/indexed-keyword (ID) fields. Spelling variants, abbreviations, and semantically equivalent expressions were merged under a preferred term. Database-indexing artefacts and nonspecific publication, demographic, study-design, or measurement descriptors were excluded. No publication records or other bibliographic fields were added, removed, or modified.

# Supplementary Table S1A. Applied synonym mappings

| **Original term** | **Harmonized term** | **Analysis** |
| --- | --- | --- |
| c reactive protein | c-reactive protein | Burst; thematic |
| CRP | c-reactive protein | Burst; thematic |
| gonarthrosis | knee osteoarthritis | Burst; thematic |
| gut microbiome | gut microbiota | Burst; thematic |
| intestinal microbiota | gut microbiota | Burst; thematic |
| IL-6 | interleukin-6 | Burst; thematic |
| knee OA | knee osteoarthritis | Burst; thematic |
| low grade inflammation | low-grade inflammation | Burst; thematic |
| mesenchymal stem cell | mesenchymal stem cells | Burst; thematic |
| MSCs | mesenchymal stem cells | Burst; thematic |
| MRI | magnetic resonance imaging | Burst; thematic |
| multiple chronic conditions | multimorbidity | Burst; thematic |
| ROS | reactive oxygen species | Burst; thematic |
| TNF-alpha | tumor necrosis factor-alpha | Burst; thematic |
| osteoarthritis (OA) | osteoarthritis | Thematic |
| osteoarthrosis | osteoarthritis | Thematic |

# Supplementary Table S1B. Applied exclusion list

| **Category** | **Excluded term** | **Analysis** |
| --- | --- | --- |
| Database/indexing artefact | priority journal | Burst; thematic |
| Database/indexing artefact | physiology | Burst; thematic |
| Nonspecific indexed condition | malignant neoplasm | Burst; thematic |
| Nonspecific measurement descriptor | protein blood level | Burst; thematic |
| Nonspecific measurement descriptor | visual analog scale | Burst; thematic |
| Publication descriptor | article | Burst; thematic |
| Publication descriptor | review | Burst; thematic |
| Publication descriptor | systematic review | Burst; thematic |
| Publication descriptor | meta-analysis / meta analysis | Burst; thematic |
| Publication descriptor | publication | Burst; thematic |
| Study-design descriptor | clinical article | Burst |
| Study-design descriptor | comparative study | Burst |
| Study-design descriptor | controlled study | Burst |
| Study-design descriptor | major clinical study | Burst |
| Demographic/indexing descriptor | human / humans | Burst; thematic |
| Demographic/indexing descriptor | male | Burst; thematic |
| Demographic/indexing descriptor | female | Burst; thematic |
| Demographic/indexing descriptor | adult / adults | Burst; thematic |
| Demographic/indexing descriptor | aged | Burst; thematic |
| Demographic/indexing descriptor | elderly | Burst; thematic |
| Demographic/indexing descriptor | older adults | Thematic |
| Demographic/indexing descriptor | animal / animals | Burst; thematic |
| Demographic/indexing descriptor | nonhuman | Burst; thematic |
| Demographic/indexing descriptor | middle aged | Burst; thematic |

# Supplementary Table S1C. Broad terms deliberately retained

| **Retained term** | **Reason for retention** |
| --- | --- |
| arthritis | Core disease concept |
| rheumatoid arthritis | Clinically meaningful disease concept |
| hand osteoarthritis | Anatomically specific osteoarthritis concept |
| pathology | Legitimate research theme |
| disease course | Legitimate clinical research theme |

# Supplementary Table S1D. Revised analysis settings

| **Analysis** | **Settings** |
| --- | --- |
| Keyword burst analysis (Figure 7) | Author Keywords and Keywords Plus; 2000-2025; one-year slices; g-index k = 25; top 20 bursts displayed |
| Thematic map (Figure 8) | Author Keywords; 198 terms; minimum cluster frequency = 5; alpha = 0.5; label-overlap avoidance enabled |

**Note.** The Analysis column records where each transformation was encountered and applied. The harmonization process affected keyword metadata only and did not alter the final set of 783 included publications.
