# Supplementary material for: Immunometabolic crosstalk in knee osteoarthritis: a bibliometric and translational analysis (2000–2025)
Source: Front Immunol. 2026 Sep 17;17:1877104. doi: 10.3389/fimmu.2026.1877104 (PMC13627379; doi:10.3389/fimmu.2026.1877104)
Supplement: Supplementary file 5 [file Table2.docx]

**Supplementary Table S2. Quantitative sensitivity analysis of the CiteSpace keyword network**

| Analysis dataset | Time span | Qualified records | Nodes (N) | Links (E) | Density | Largest CC, nodes (% of pre-pruning network) | Modularity Q | Mean silhouette | No. of clusters |
| --- | --- | --- | --- | --- | --- | --- | --- | --- | --- |
| Full merged dataset (primary analysis) | 2000–2025 | 783 | 544 | 3,748 | 0.0254 | 544 (87.32%) | 0.4999 | 0.6836 | 11 |
| Full merged dataset excluding 2025 | 2000–2024 | 671 | 509 | 3,384 | 0.0262 | 509 (87.01%) | 0.4825 | 0.6774 | 11 |
| Article-only subset | 2000–2025 | 632 | 546 | 3,620 | 0.0243 | 546 (89.95%) | 0.5172 | 0.7058 | 10 |
| Web of Science-only subset | 2002–2025 | 422 | 97 | 165 | 0.0354 | 97 (35.66%) | 0.8115 | 0.9743 | 7 |

**Note.** All analyses used one-year time slices, keyword nodes, cosine link strength, the g-index (k = 25), and Pathfinder pruning. Cluster labels were generated using the log-likelihood ratio method.

For the Web of Science-only sensitivity analysis, 423 records were initially retrieved and 422 qualified records were analyzed by CiteSpace; no eligible records were available for 2000–2001.

**Interpretation.** Network size, density, modularity, and silhouette values were similar after excluding 2025 or restricting the dataset to articles. The Web of Science-only network was smaller and more fragmented, reflecting reduced database coverage.

**Top-keyword stability across sensitivity analyses**

| Analysis dataset | Top 10 harmonized keywords (ranked by occurrence) | Overlap with primary top 10 |
| --- | --- | --- |
| Full merged dataset (primary analysis) | knee osteoarthritis; osteoarthritis; inflammation; obesity; pain; metabolic disorder; body mass; knee; c-reactive protein; metabolism | Reference |
| Full merged dataset excluding 2025 | knee osteoarthritis; osteoarthritis; inflammation; obesity; pain; metabolic disorder; body mass; metabolic syndrome; cartilage; knee | 8/10 (80%) |
| Article-only subset | knee osteoarthritis; osteoarthritis; inflammation; obesity; metabolic disorder; pain; body mass; knee; c-reactive protein; metabolism | 10/10 (100%) |
| Web of Science-only subset | knee osteoarthritis; osteoarthritis; inflammation; obesity; pain; metabolic syndrome; cartilage; low-grade inflammation; association; synovial fluid | 5/10 (50%) |

**Note.** Keywords were ranked by document-level occurrence in the Author Keywords and Keywords Plus fields after applying the synonym and stop-list rules reported in Table S1. “Middle aged” was treated as a demographic/indexing descriptor under the same exclusion rule as “adult” and “aged.”

**Overall conclusion.** The primary inflammation–metabolism–KOA pattern remained when we excluded 2025 records or non-article records. Database restriction had a larger effect on network size and peripheral keyword rankings, but the leading concepts—knee osteoarthritis, osteoarthritis, inflammation, obesity, and pain—remained unchanged.
